# Neoadjuvant atezolizumab for resectable non-small cell lung cancer: an open-label, single-arm phase II trial

**DOI:** 10.1038/s41591-022-01962-5

**Published:** 2022-09-12

**Authors:** Jamie E. Chaft, Filiz Oezkan, Mark G. Kris, Paul A. Bunn, Ignacio I. Wistuba, David J. Kwiatkowski, Dwight H. Owen, Yan Tang, Bruce E. Johnson, Jay M. Lee, Gerard Lozanski, Maciej Pietrzak, Michal Seweryn, Woo Yul Byun, Katja Schulze, Alan Nicholas, Ann Johnson, Jessica Grindheim, Stephanie Hilz, David S. Shames, Chris Rivard, Eric Toloza, Eric B. Haura, Ciaran J. McNamee, G. Alexander Patterson, Saiama N. Waqar, Valerie W. Rusch, David P. Carbone, LCMC study investigators, Saiama N. Waqar, Elaine Shum, Misako Nagasaka, Marianna Koczywas, Edward B. Garon, David J. Finley, David R. Camidge, Jennifer W. Carlisle, Justin D. Blasberg

**Affiliations:** 1 Memorial Sloan Kettering Cancer Center, New York, NY USA; 2 Weill Cornell Medical College, New York, NY USA; 3 The Ohio State University Comprehensive Cancer Center, Columbus, OH USA; 4 University Medicine Essen, Ruhrlandklinik, Department of Interventional Pulmonology, University Duisburg-Essen, Essen, Germany; 5 German Cancer Research Center (DKFZ), A420, Heidelberg, Germany; 6 Fifth Medical Department, Section of Pulmonology, Faculty of the University of Heidelberg, University Medicine Mannheim, Mannheim, Germany; 7 University of Colorado School of Medicine, Aurora, CO USA; 8 The University of Texas MD Anderson Cancer Center, Houston, TX USA; 9 Dana-Farber Cancer Institute, Boston, MA USA; 10 Brigham and Women’s Hospital, Boston, MA USA; 11 The Ohio State University Wexner Medical Center, Columbus, OH USA; 12 David Geffen School of Medicine at UCLA, Los Angeles, CA USA; 13 Biobank Lab, Department of Molecular Biophysics, University of Lodz, Lodz, Poland; 14 Centre for Data Analysis, Modeling and Computational Sciences, University of Lodz, Lodz, Poland; 15 Genentech, Inc., South San Francisco, CA USA; 16 Moffitt Cancer Center and Research Institute, Tampa, FL USA; 17 Washington University School of Medicine, St. Louis, MO USA; 18 Pelotonia Institute for Immuno-Oncology, Columbus, OH USA; 19 Alvin J. Siteman Cancer Center, St. Louis, MO USA; 20 New York University Langone Health, New York, NY USA; 21 Perlmutter Comprehensive Cancer Center, New York University, New York, NY USA; 22 Karmanos Cancer Institute, Detroit, MI USA; 23 City of Hope Comprehensive Cancer Center, Duarte, CA USA; 24 Dartmouth-Hitchcock Medical Center, Lebanon, NH USA; 25 University of Colorado Cancer Center, Aurora, CO USA; 26 Winship Cancer Institute, Emory University School of Medicine, Atlanta, GA USA; 27 Yale University School of Medicine, New Haven, CT USA

**Keywords:** Non-small-cell lung cancer, Phase II trials

## Abstract

In an ongoing, open-label, single-arm phase II study (NCT02927301), 181 patients with untreated, resectable, stage IB–IIIB non-small cell lung cancer received two doses of neoadjuvant atezolizumab monotherapy. The primary end point was major pathological response (MPR; ≤10% viable malignant cells) in resected tumors without *EGFR* or *ALK* alterations. Of the 143 patients in the primary end point analysis, the MPR was 20% (95% confidence interval, 14–28%). With a minimum duration of follow-up of 3 years, the 3-year survival rate of 80% was encouraging. The most common adverse events during the neoadjuvant phase were fatigue (39%, 71 of 181) and procedural pain (29%, 53 of 181), along with expected immune-related toxicities; there were no unexpected safety signals. In exploratory analyses, MPR was predicted using the pre-treatment peripheral blood immunophenotype based on 14 immune cell subsets. Immune cell subsets predictive of MPR in the peripheral blood were also identified in the tumor microenvironment and were associated with MPR. This study of neoadjuvant atezolizumab in a large cohort of patients with resectable non-small cell lung cancer was safe and met its primary end point of MPR ≥ 15%. Data from this single-arm, non-randomized trial suggest that profiles of innate immune cells in pre-treatment peripheral blood may predict pathological response after neoadjuvant atezolizumab, but additional studies are needed to determine whether these profiles can inform patient selection and new therapeutic approaches.

## Abstract (web summary)

In a single-arm, non-randomized trial, neoadjuvant atezolizumab therapy in a large cohort of patients with resectable non-small cell lung cancer was safe and the study met its primary end point of major pathological response ≥15%.

## Main

The survival of patients with resectable non-small cell lung cancer (NSCLC) has not substantially improved since the establishment of adjuvant chemotherapy as standard treatment more than 20 years ago. Inhibitors of PD-1 or PD-L1 are approved for the treatment of advanced-stage NSCLC and resected stage II–III, PD-L1-expressing NSCLC^1^. These agents have shown some benefit when given before surgery in small (*n* = 21–23) studies of patients with resectable NSCLC; however, the pathological response rates in these studies have wide confidence intervals (CIs) and predictive biomarkers of response remain unclear^2,3^. The phase II Lung Cancer Mutation Consortium 3 (LCMC3) study was performed to evaluate the efficacy of neoadjuvant atezolizumab, a PD-L1 inhibitor, in a large population of treatment-naive patients with resectable, stage IB–IIIB NSCLC. Prospective correlative studies were performed to elucidate potential predictors of treatment response and resistance.

**Extended Data Fig. 1:**
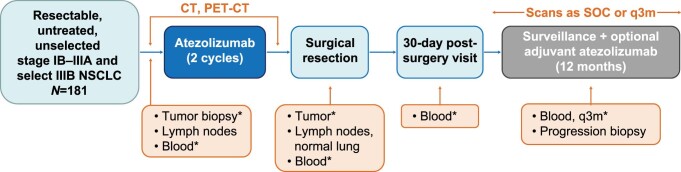
Study design. *Mandatory. CT, computed tomography; NSCLC, non-small cell lung cancer; PET, positron emission tomography; q3m, every 3 months; SOC, standard of care.

Atezolizumab is hypothesized to enhance antitumor immunity by restoring the function of cytotoxic T cells^4^, but the details of this mechanism in humans and the effects of atezolizumab on other immune cell populations are largely undefined. Because not all patients respond to PD-(L)1 inhibition, identifying biomarkers predictive of response and resistance may aid in treatment selection. Moreover, elucidating the mechanisms by which cancer cells evade antitumor immunity may inform rational combination therapies. To define the clinical and biological effects of neoadjuvant atezolizumab and to identify biomarkers predictive of response (or lack thereof), the LCMC3 study evaluated the immune environment pre- and post-treatment with atezolizumab and correlated these changes with the primary efficacy measure of MPR (Extended Data Fig. 1).

## Results

### Patients

**Fig. 1:**
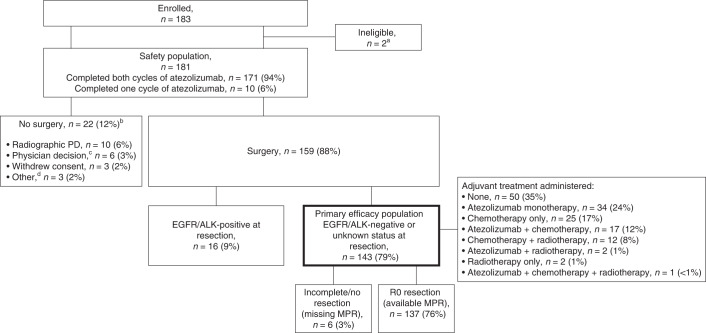
Patient disposition. Primary efficacy population is bolded. ^a^Two patients were determined to have hemangioma and solitary fibrous tumor at resection despite initial pathology consistent with NSCLC. ^b^Includes one EGFR-positive patient. ^c^The reasons were clinical progression (*n* = 3), physician did not want to delay patient surgery (*n* = 1), physician did not consider the patient a good surgical candidate (*n* = 1) and physician discontinued patient from the study because of an AE (*n* = 1). ^d^One patient was determined to have pre-existing congestive heart failure, one declined surgery and one was lost to follow-up.

Between 20 April 2017 and 3 February 2020, 181 patients with NSCLC were enrolled (Fig. 1). Baseline demographics and disease characteristics are summarized in Table 1 and Supplementary Table 1. A total of 171 (94%) patients received both doses of atezolizumab; 10 (6%) did not (due to 7 adverse events [AEs], 2 physician decisions and 1 withdrawal). The seven AEs leading to discontinuation of atezolizumab were infusion-related reaction (*n* = 2), pyrexia (*n* = 2), fatigue, diverticulitis and dyspnea (all *n* = 1). During the second infusion, 13 patients required dose interruption, 12 because of infusion-related reactions and 1 because of ongoing (non-worsening) cough and dyspnea. Of the 181 patients, 159 (88%) had surgery with curative intent; 22 (12%) did not have surgery, as detailed in Fig. 1.

**Table 1:** Baseline demographics and disease characteristics

	Patients (*n* = 181)
Median age, years (range)	65.0 (37–83)
Female, *n* (%)	93 (51)
Race, *n* (%)	
White	145 (81)
Black/African American	13 (7)
Asian	9 (5)
Unknown	12 (7)
ECOG performance status score, *n* (%)	
0	104 (57)
1	77 (43)
Clinical stage, *n* (%)	
IB	18 (10)
IIA	16 (9)
IIB	55 (30)
IIIA	70 (39)
IIIB^a^	22 (12)
Histology, *n* (%)	
Non-squamous	112 (62)
Squamous	69 (38)
History of tobacco use, *n* (%)	
Never	18 (10)
Current	35 (19)
Former	128 (71)
Median pack-years, *n* (range)	22.75 (0–162.0)
PD-L1 TPS, *n* (%)^b^	
<1%	69 (38)
1–49%	28 (15)
≥50%	49 (27)
Unknown^c^	35 (19)
*EGFR* mutation, *n* (%)^d^	
Positive	11 (6)
Negative	154 (85)
Unknown^e^	16 (9)
*ALK* rearrangement, *n* (%)^d^	
Positive	6 (3)
Negative	162 (90)
Unknown^f^	13 (7)

ALK, anaplastic lymphoma kinase; ECOG, Eastern Cooperative Oncology Group; EGFR, epidermal growth factor receptor; PD-L1, programmed death-ligand 1; TPS, tumor proportion score.

^a^Select IIIB includes T3N2 or T4 (by size criteria, not by mediastinal invasion), per the American Joint Committee on Cancer Staging System (8th edition). ^b^PD-L1 status was centrally determined by immunohistochemistry using the DAKO PD-L1 (22C3) assay. ^c^The large number of patients with ‘unknown’ PD-L1 status was attributable to missing samples and failed testing. ^d^Determined either locally or centrally from screening tissue (when adequate) or resected tumor tissue. ^e^*EGFR* status was unknown in 16 patients (non-squamous, *n* = 5; squamous, *n* = 11). ^f^*ALK* rearrangement status was unknown in 13 patients (non-squamous, *n* = 5; squamous, *n* = 8).

### Efficacy

**Fig. 2:**
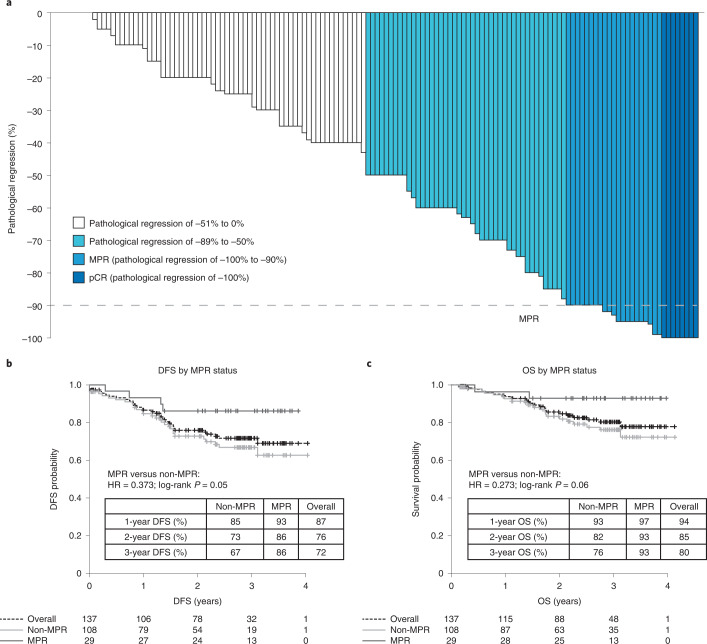
Clinical outcomes in patients who had surgical resection and whose tumors did not have known *EGFR* or *ALK* alterations. **a**, Pathological response (*n* = 143). Pathological regression is defined as percentage viable tumor cells – 100%. **b**, DFS by MPR status in patients with R0 resections (*n* = 137). **c**, OS by MPR status in patients with R0 resections (*n* = 137). HR, hazard ratio.

Of the 159 patients who had surgery, 16 (10%) had tumors harboring *EGFR* mutations (exon 19 deletion, *n* = 8; exon 20 insertion, *n* = 1; L858R, *n* = 1) or *ALK* rearrangements (*n* = 6) and were excluded from the primary efficacy analysis. In the primary analysis population, the MPR rate was 20% (29 of 143; 95% CI 14–28%) and the pathological complete response (pCR) rate was 6% (8 of 143; 95% CI 3–11%) (Fig. 2a). The characteristics of patients with pCR are summarized in Supplementary Table 2. In subgroup analyses, the odds of MPR were higher in patients who were female or who had partial response as per Response Evaluation Criteria in Solid Tumors (RECIST) criteria, N1 disease or squamous histology (Supplementary Table 3). The secondary end point of MPR rate in patients with a PD-L1 tumor proportion score (TPS) of <1%, 1–49% and ≥50% at screening was 11% (6 of 53), 5% (1 of 20) and 33% (15 of 45), respectively (*P* = 0.01, two-sided Fisher’s exact test). In an analysis of data from 111 patients, baseline PD-L1 TPS was found to correlate significantly with pathological response (*R* = −0.37; *P* < 0.001). No tumor with *EGFR* or *ALK* alterations demonstrated radiographical response or MPR. Regarding radiographical responses in the 181 patients who received ≥1 dose of atezolizumab, 11 had partial responses (6%), 147 (81%) had stable disease, 13 (7%) had progressive disease (PD; local, *n* = 6; regional, *n* = 7) and 10 (6%) were not assessed.

**Extended Data Fig. 2:**
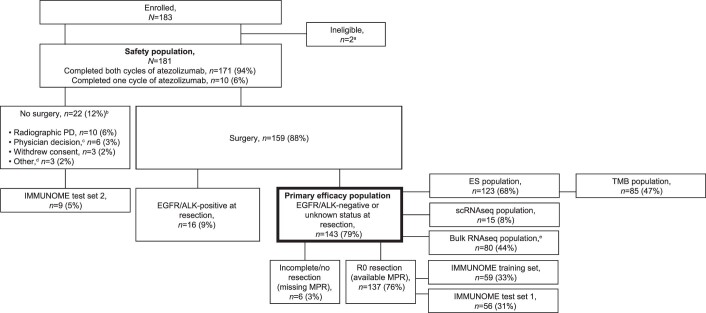
CONSORT diagram for the various biomarker analyses. ^a^Two patients were determined to have hemangioma and solitary fibrous tumor at resection despite initial pathology consistent with NSCLC. ^b^Includes 1 EGFR-positive patient. ^c^The reasons were as follows: clinical progression (*n* = 3), physician did not want to delay patient surgery (*n* = 1), physician did not consider the patient a good surgical candidate (*n* = 1), and physician discontinued patient from the study because of an adverse event (*n* = 1). ^d^One patient was determined to have preexisting congestive heart failure, 1 declined surgery, and 1 was lost to follow-up. ^e^Eighty patients provided a total of 98 samples for the bulk RNA-seq analyses (54 at baseline and 44 at surgery). ALK, anaplastic lymphoma kinase; EGFR, epidermal growth factor receptor; ES, exome sequencing; MPR, major pathologic response; NSCLC, non-small cell lung cancer; PD, progressive disease; RECIST, Response Evaluation Criteria in Solid Tumors; RNA-seq, ribonucleic acid sequencing; scRNA-seq, single-cell ribonucleic acid sequencing; TMB, tumor mutational burden.

**Extended Data Fig. 3:**
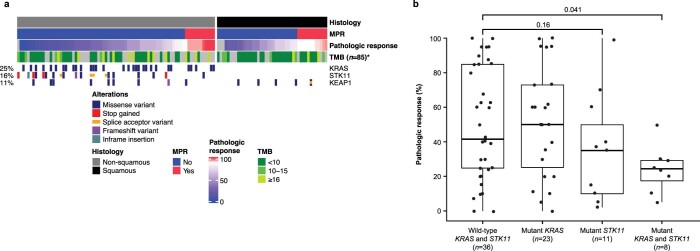
Relationship between MPR, histology, and TMB. **(a)** Pathologic response by mutation status (*n* = 85). TMB showed a positive but not significant trend with pathologic response in non-squamous (R = 0.28; *P* = 0.05) and squamous (R = 0.23; *P* = 0.22) tumors. *KEAP1* mutations were not significantly associated with pathologic response in either non-squamous (*P* = 0.46) or squamous tumors (*P* = 0.98). *KRAS* and *STK11* mutations were found only in non-squamous tumors, where mutations in *STK11* were found to significantly associate with pathologic response (*P* = 0.01), and mutations in *KRAS* were not (*P* = 0.87). **(b)** Pathologic response by the overlapping mutational status of *KRAS* and *STK11* in patients with non-squamous NSCLC (*n* = 78). The maximum and minimum values of the boxes denote the IQR. The line within the IQR denotes the median. The extremities of the dashed lines represent the 5^th^ to 95^th^ percentiles. *TMB was only determined for the subset of patients with ES data from baseline and/or surgery with tumor purity ≥15%. *P* values for TMB in Panel a were determined via linear correlation test (Pearson). *P* values for mutation status in Panels a and b were determined via two-sided Wilcoxon rank sum test. ES, exome sequencing; IQR, interquartile range; KEAP1, Kelch-like ECH-associated protein 1; KRAS, Kirsten rat sarcoma viral oncogene homolog; MPR, major pathologic response; NS, non-significant; NSCLC, non-small cell lung cancer; STK11, serine/threonine kinase 11; TMB, tumor mutational burden.

Exome sequencing data from analysis of pre-treatment paired tumor and normal samples were available for 123 patients (44 squamous and 79 non-squamous) (Extended Data Fig. 2). The secondary end point of MPR rate in patients with tumor mutational burden <10, 10–15 and ≥16 mutations per Mb was 13% (8 of 60), 10% (1 of 10) and 33% (5 of 15), respectively (<10 versus 10–15, *P* = 1.00; <10 versus ≥16, *P* = 0.12; 10–15 versus ≥16, *P* = 0.34, two-sided Fisher’s exact test) (Extended Data Fig. 3a). *STK11* mutations demonstrated a trend toward lesser pathological regression versus wild-type *STK11*, significantly in patients with co-mutation of *KRAS* and *STK11* (Extended Data Fig. 3b). *KEAP1* mutations were not associated with pathological response.

**Extended Data Fig. 4:**
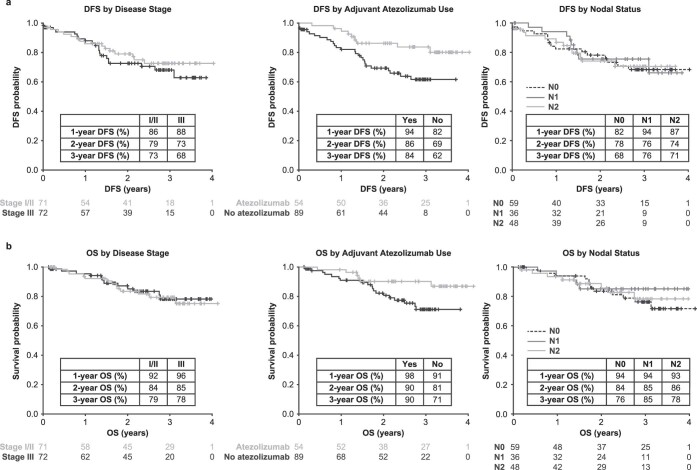
DFS (a) and OS (b) by disease stage, use of adjuvant atezolizumab, and lymph node status (*n* = 143). The MPR rate in patients who did and did not receive adjuvant atezolizumab was 41% (22/54) and 8% (7/89), respectively. DFS, disease-free survival; OS, overall survival.

Adjuvant treatment is summarized in Fig. 1. The exploratory end points of median disease-free survival (DFS) and overall survival (OS) were not reached. The 3-year DFS and OS were 72% (95% CI 62–79%) and 80% (95% CI 71–87%), respectively. Survival by MPR status is presented in Fig. 2b,c. Survival by disease stage, adjuvant atezolizumab use and lymph node status is presented in Extended Data Fig. 4. The duration of DFS in the eight patients with pCR is presented in Supplementary Table 2.

### Safety

Of the 181 patients in the safety-evaluable population, 97% experienced at least one AE during the neoadjuvant phase (up to 90 d after last dose of neoadjuvant atezolizumab), most commonly fatigue (39%) and procedural pain (29%) (Supplementary Table 4). The most frequent atezolizumab-related AE was fatigue (20%). Immune-related AEs were reported in 75 (41%) patients, most commonly increases in aspartate aminotransferase (9%, *n* = 16), alanine aminotransferase (8%, *n* = 15), maculopapular rash (8%*, n* = 15) and infusion-related reaction (8%, *n* = 15). Among the 20 (11%) patients with a treatment-related grade ≥3 AE, pneumonitis (2%, *n* = 4) and pneumonia (2%, *n* = 3) were the most frequent. Three (2%) patients died during the neoadjuvant phase; only 1 death was treatment-related (pneumonitis) (Supplementary Table 4).

### IMMUNOME

**Fig. 3:**
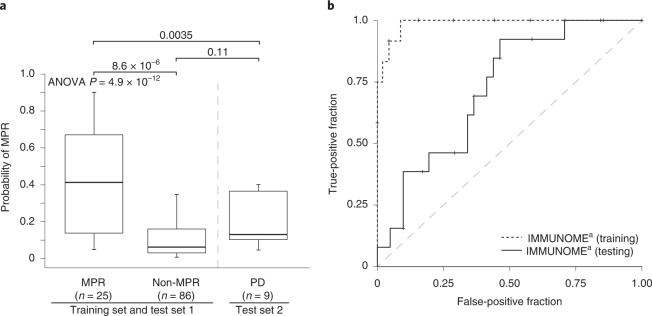
Performance of GAM–LASSO MPR predictive models. **a**, Use of the GAM–LASSO model to predict MPR in test set 2, which consisted of patients within LCMC3 who were not included in either the training set (*n* = 57) or test set 1 (*n* = 54). MPR was not assessed in these nine patients because of no resection. The MPR and non-MPR cohorts derived from the merge of the model’s training set and test set 1. The maximum and minimum values of the boxes denote the IQR. The line within the IQR denotes the median. The extremities of the dashed lines represent the minimum and maximum values of the data, which are 1.5× below the first quartile and 1.5× above the third quartile. The parameters for null hypothesis testing via analysis of variance (ANOVA) were as follows: d.f. = 2, total sum of squares = 1.976, mean squares = 0.988, *F*-value = 32.799 and Pr(>F) = 4.914 × 10^−12^. The statistical details for the comparison of MPR and non-MPR were *t* = −5.47, d.f. = 27.02, two-sided *P* = 8.6 × 10^−6^ and 95% CI = −0.439 to −0.200. The statistical details for the comparison of MPR and PD were *t* = −3.18, d.f. = 28.45, two-sided *P* = 0.0035 and 95% CI = −0.383 to −0.083. The statistical details for the comparison of non-MPR and PD were *t* = −1.77, d.f. = 9.52, two-sided *P* = 0.11 and 95% CI = −0.195 to 0.023. No adjustment was made for multiplicity. **b**, ROC curves for the training set and test set 1. The dashed *y* = *x* line, which represents random assignment, is included for reference. ^a^Immunophenotyping via flow cytometry. IQR, interquartile range; ROC, receiver operating characteristic.

**Extended Data Fig. 5:**
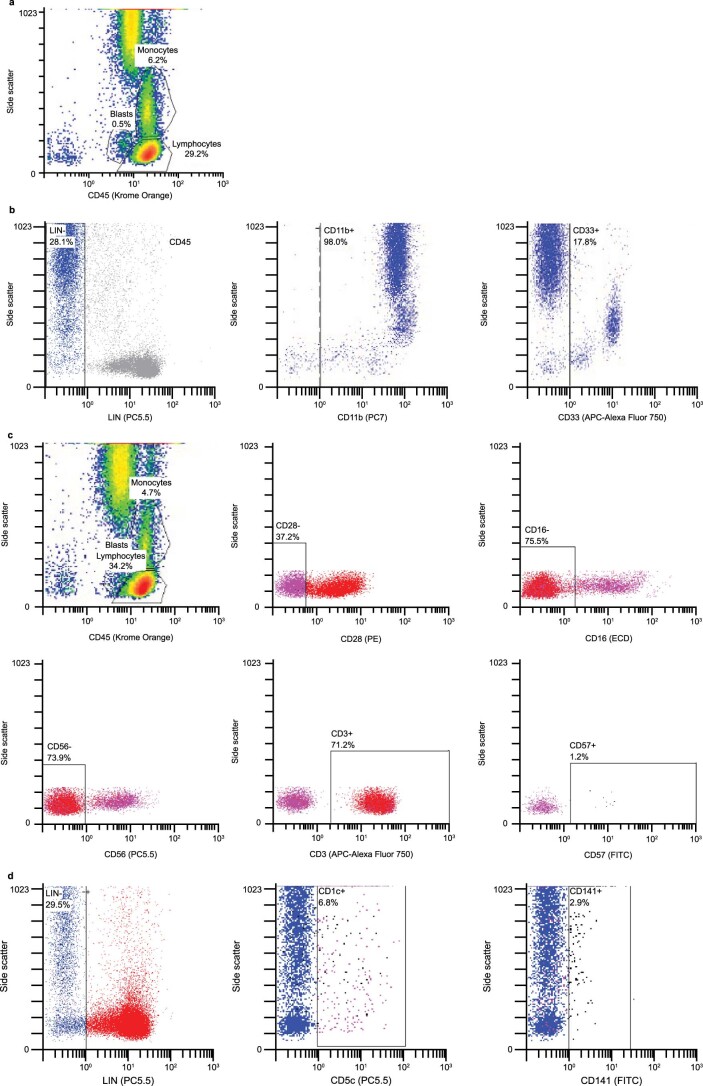
Gating strategy for the IMMUNOME tubes described in Supplementary Table 5. **(a)** Tubes 1–11 were gated on the basis of CD45 and side scatter. The immune cell populations of interest were those falling within the region ‘lymphocytes,’ which was defined by CD45^bright^, a low side scatter, and a lymphosum (CD3^+^ CD19^+^ CD56^+^ CD16^+^) totaling 95%–100%, with <5% myeloid contamination. Tube 1 (lymphosum) contained lymphosum markers, as well as myeloid markers (CD13/CD14). The gate established for Tube 1 was applied to Tubes 2–11. **(b)** Tube 12 (myeloid cells) used LIN (CD3^+^ CD19^+^ CD56^+^) as an exclusion gate. Plots were gated using three different strategies: LIN^−^ total, LIN^−^ CD11b^+^, and LIN^−^ CD33^+^. **(c)** Tube 13 (senescent cells) used both lymphocyte and sequential gating to find the immune cell population of interest. Lymphocyte gating isolated CD28^−^ CD16^−^ CD56^−^ CD3^+^ cells, which were sequentially gated on CD57. Events falling with the CD57^+^ region were considered senescent cells. Senescent cells were further classified into subsets defined by positivity and negativity for CD4, CD8, KLRG1, and CD127. **(d)** Tube 14 (dendritic cells) used LIN as an exclusion gate. Plots were gated using three different strategies: LIN^−^ total, LIN^−^ CD1c^+^, and LIN^−^ CD141^+^. CD, cluster of differentiation; KLRG1, killer cell lectin-like receptor subfamily G member 1; LIN, lineage.

**Extended Data Fig. 6:**
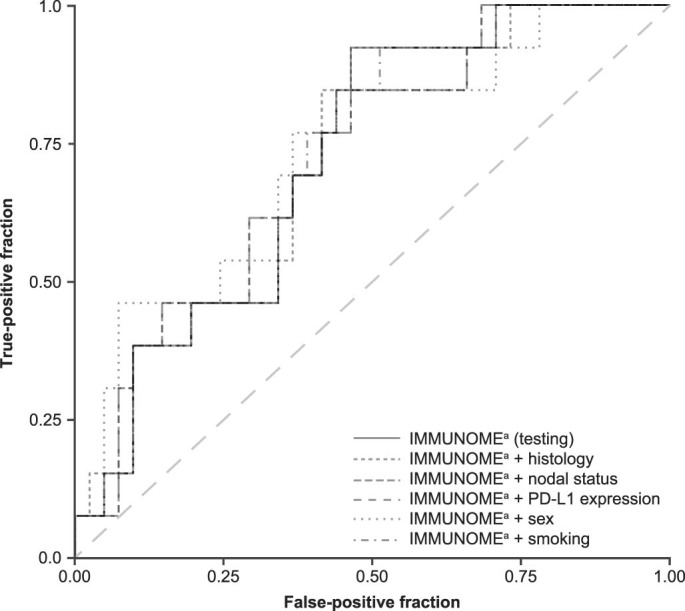
ROC curves for test set 1 (*n* = 54), which considered only peripheral blood IMMUNOME, as well as for test set 1 in combination with histology (non-squamous [*n* = 32] vs. squamous [*n* = 22]), nodal status (N1/N2 [*n* = 35] vs. N0 [*n* = 19]), PD-L1 expression (*n* = 54), sex (female [*n* = 26] vs. male [*n* = 28]), or smoking status/history (never [*n* = 6] vs. current/former [*n* = 48]). The dashed y = x line, which represents random assignment, is included for reference. ^a^Immunophenotyping via flow cytometry. PD-L1, programmed death-ligand 1; ROC, receiver operating characteristic.

In exploratory analyses, 111 pre-treatment peripheral blood samples were evaluated via ten-color 60-marker flow cytometry (IMMUNOME) (Supplementary Table 5 and Extended Data Fig. 5). Samples were split into a training set (*n* = 57) to develop a predictive model for MPR and a test set 1 (*n* = 54) (Extended Data Fig. 2). The area under the curve (AUC) was 0.987 for the training set and 0.722 for test set 1; the addition of individual clinical parameters did not significantly improve the predictive power (Fig. 3, Extended Data Fig. 6 and Supplementary Table 6). The final multiparametric GAM–LASSO (generalized additive model–least absolute shrinkage and selection operator) model consisted of 14 immune cell subsets in the pre-treatment peripheral blood that significantly correlated with MPR. Higher prevalence of non-T/non-natural killer (NK) cells expressing the immunoregulatory receptors immunoglobulin-like transcript 2 (ILT2), killer cell immunoglobulin-like receptor (KIR) 2DL1 (KIR2DL1) and KIR2DL2 and of NK group 2 member D (NKG2D)^+^ non-T/non-NK cells positively associated with MPR (Supplementary Table 7 details immune cell descriptions and effect sizes). Higher prevalence of NK and NK-like T cell subsets in the peripheral blood (several of which express inhibitory receptors such as ILT2, NKG2A and NKG2D), subsets of γ/δ T cells, γ/δ NK-like T cells, degranulated myeloid cells and naive CD4^+^/CD8^+^ T cells inversely associated with MPR (Supplementary Table 7).

The ability of the final model to predict the probability of MPR was evaluated in test set 2, consisting of nine study participants with radiographical PD who were not included in the training set or test set 1. The mean probability of MPR was predicted to be 0.20 in the PD cohort, 0.11 in the non-MPR cohort and 0.43 in the MPR cohort (PD versus non-MPR, *P* = 0.11; PD versus MPR, *P* = 0.0035; non-MPR versus MPR, *P* < 0.0001) (Fig. 3 and Supplementary Table 8).

**Extended Data Fig. 7:**
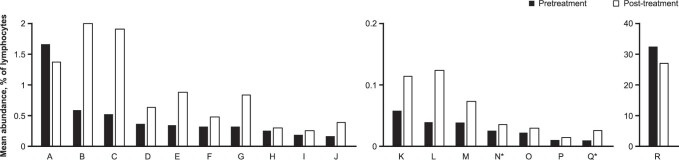
Immune cell subsets with significant (two-sided *p* < 0.05) changes in abundance before (baseline) and after (at surgery) neoadjuvant treatment with atezolizumab. No adjustment was made for multiplicity. **A**, CD45^+^ CD62L^+^ CD27^+^ CD56/16^−^ CD45RO^+^ CCR7^−^ CD45RA^−^ CD4^−^ CD3^+^ CD8^+^ (*p* = 0.0081); **B**, CD45^+^ CD33^+^ HLA-DR^+^ CD124^−^ CD14^+^ CD11b^+^ CD66b^−^ CD16^−^ CD33^+^ CD15^+^ (*p* = 0.0042); **C**, CD45^+^ HLA-DR^+^ CD124^−^ CD14^+^ CD11b^+^ CD66b^−^ CD16^−^ CD33^+^ CD15^+^ (*p* = 0.0042); **D**, CD45^+^ CD94^+^ NKG2D^+^ CD3^−^ CD56^+^ CD117^−^ NKG2A^+^ CD127^−^ CD161^−^ CD16^+^ (*p* = 0.0036); **E**, CD45^+^ LIN^+^ HLA-DR^+^ CD33^+^ CD16^+^ CD11b^+^ CD15^+^ (*p* = 0.0092); **F**, CD45^+^ HLA-DR^−^ CD69^+^ CD19^−^ CD56^+^ CD16^−^ CD134^−^ CD4^−^ CD3^+^ CD8^+^ (*p* = 0.0079); **G**, CD45^+^ CD11b^+^ HLA-DR^+^ CD124^−^ CD14^+^ CD11b^+^ CD66b^−^ CD16^−^ CD33^+^ CD15^+^ (*p* = 0.0157); **H**, CD45^+^ γ/δ^-^ α/β^+^ CD19^−^ CD56^+^ CD16^−^ CD13/14^−^ CD4^+^ CD3^+^ CD8^−^ (*p* = 0.0113); **I**, CD45^+^ HLA-DR^−^ CD69^+^ CD3^+^ KIR3DL1^−^ KIR2DL2^−^ NKG2A^−^ CD56^+^ KIR2DL1^−^ CD16^−^ (*p* < 0.0001); **J**, CD45^+^ CD107a/b^-^ CD159c^+^ CD3^−^ KIR3DL1^−^ KIR2DL2^−^ NKp80^+^ CD56^+^ KIR2DL1^−^ CD16^+^ (*p* = 0.0025); **K**, CD45^+^ HLA-DR^−^ CD69^+^ CD19^−^ CD56^+^ CD16^−^ CD134^−^ CD4^−^ CD3^+^ CD8^−^ (*p* = 0.0004); **L**, CD45^+^ CD16^+^ CD336^−^ CD3^−^ CD244^+^ CD335^+^ NKG2D^+^ CD56^−^ CD161^+^ CD337^−^ (*p* = 0.0002); **M**, CD45^+^ γ/δ^-^ α/β^+^ CD19^−^ CD56^+^ CD16^−^ CD13/14^−^ CD4^−^ CD3^+^ CD8^−^ (*p* = 0.0191); **N**, CD45^+^ HLA-DR^−^ CD69^+^ CD3^+^ KIR3DL1^−^ KIR2DL2^−^ NKG2A^+^ CD56^+^ KIR2DL1^−^ CD16^−^ (*p* = 0.0307); **O**, CD45^+^ CD62L^−^ CD27^+^ CD56/16^+^ CD45RO^−^ CCR7^−^ CD45RA^+^ CD4^−^ CD3^+^ CD8^+^ (*p* = 0.0400); **P**, CD45^+^ CD94^−^ NKG2D^−^ CD3^+^ CD56^+^ CD117^−^ NKG2A^−^ CD127^+^ CD161^+^ CD16^+^ (*p* = 0.0480); **Q**, CD45^+^ HLA-DR^+^ CD69^+^ CD3^−^ KIR3DL1^−^ KIR2DL2^−^ NKG2A^+^ CD56^−^ KIR2DL1^−^ CD16^+^ (*p* = 0.0014); **R**, CD45^+^ LIN^−^ HLA-DR^−^ CD33^−^ CD16^+^ CD11b^+^ CD15^+^ (p = 0.0030). *Pre-treatment predictor of MPR. LIN included CD19, CD3, and CD56. NKG2A is also known as CD159a, NKG2D as CD314 and KLRK1, KIR2DL1 as CD158a, KIR2DL2 as CD158b, CD335 as NKp46, and CD337 as NKp30. α/β, α/β chains of the T cell receptor; γ/δ, γ/δ chains of the T cell receptor; CCR7, C-C motif chemokine receptor 7; CD, cluster of differentiation; HLA, human leukocyte antigen; KIR, killer cell immunoglobulin-like receptor; KLR, killer cell lectin-like receptor; LIN, lineage; MPR, major pathologic response; NK, natural killer; NKG2, natural killer group protein 2.

When comparing pre- and post-atezolizumab peripheral blood, non-T/non-NK cells expressing ILT2, KIR2DL1 and KIR2DL2, as well as NKG2D^−^ NKG2A^+^ CD94^+^ CD127^−^ CD161^+^ NK-like T cells, activated CD8^+^ T cells, activated CD56^−^/CD16^−^ NK cells and NKG2A^+^ NKG2D^+^ CD127^+^ T cells underwent significant expansion (Extended Data Fig. 7). NKG2D^+^ NKG2A^+^ CD94^+^ CD127^+^ CD161^−^ NK-like T cells, central memory CD4^+^ T cells, a subset of immature myeloid lineage cells and ILT2^+^ NKG2A^+^ KIR2DL1^+^ NK cells underwent significant contraction in atezolizumab-treated patients who experienced MPR. A predictive model using pre- and post-treatment peripheral blood samples was calculated, with an AUC in test set 1 of 0.726 (Supplementary Table 9).

### Gene expression analysis of tumor tissue

**Fig. 4:**
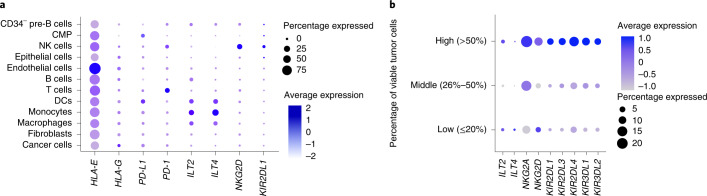
scRNA-seq analysis of selected genes expressed in tumor tissue from 15 patients following treatment with neoadjuvant atezolizumab. **a**, The expression of different NK cell surface receptors was determined by scRNA-seq. **b**, Tumor samples collected at resection were classified into three groups of five samples each on the basis of the percentage of viable tumor cells by pathological analysis: low (≤25% viable tumor cells), middle (26–50%) and high (>50%). Dot size represents the percentage of NK cells in the group expressing the gene. Color represents the scaled average normalized expression. NK cells were downsampled to have the same number of cells in each group. ILT2 is also known as LILRB1, ILT4 as LILRB2, KIR2DL1 as CD158a, NKG2D as CD314 and KLRK1 and PD-L1 as CD274. CD, cluster of differentiation; CMP, common myeloid progenitor; ILT, immunoglobulin-like transcript; KIR2DL1, killer cell immunoglobulin-like receptor DL1; LILRB, leukocyte immunoglobulin-like receptor subfamily B; NKG2, natural killer group protein 2.

**Extended Data Fig. 8:**
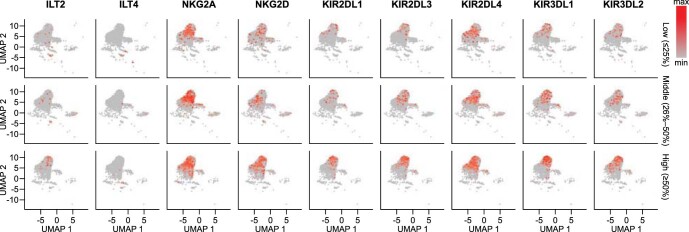
Association between gene expression in NK cells and the percentage of viable tumor cells. The expression of different NK cell surface receptors was determined by scRNA-seq. Tumor samples collected at resection were classified into 3 groups of 5 samples each based on the percentage of viable tumor cells by pathologic analysis: low (≤25% viable tumor cells), middle (26%–50%), and high (>50%). UMAP plots of NK cells show normalized expression of several receptors in the 3 groups of viable tumor cell numbers. NK cells were down-sampled to have the same number of cells in each group. ILT2 is also known as LILRB1, ILT4 as LILRB2, NKG2A as CD159a, NKG2D as CD314 and KLRK1, KIR2DL1 as CD158a, KIR2DL3 as CD158b, KIR2DL4 as CD158d, KIR3DL1 as CD158e1, and KIR3DL2 as CD158k. CD, cluster of differentiation; ILT, immunoglobulin-like transcript; KIR, killer cell immunoglobulin-like receptor; KLR, killer cell lectin-like receptor; LILRB, leukocyte immunoglobulin-like receptor subfamily B; max, maximum; min, minimum; NK, natural killer; NKG2, natural killer group protein 2; scRNA-seq, single-cell ribonucleic acid sequencing; UMAP, uniform manifold approximation and projection.

**Extended Data Fig. 9:**
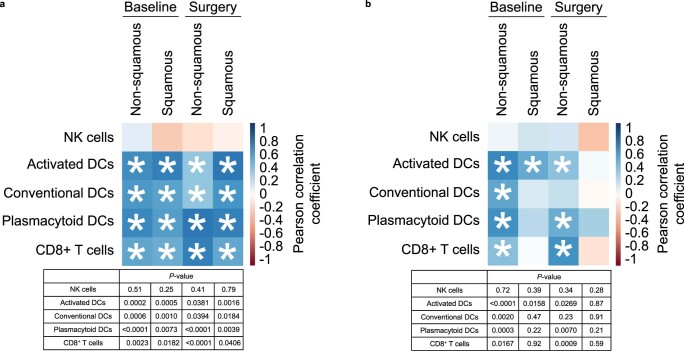
Association of (a) *ILT2* and (b) *PD*^*−*^*L1* transcripts with the cell type enrichment scores of specific immune cell subsets derived from bulk RNA-seq at baseline and at surgery by tumor histology. Relative immune cell abundance was estimated from RNA-seq data using the cell type enrichment analysis tool xCell and correlated with the abundance (log_2_ cpm) of *ILT2* or *PD-L1*. Results are shown for all baseline and surgical samples with RNA-Seq, split by histology. A positive correlation coefficient (blue) indicates samples with an increased abundance of a given immune cell signature also having an increased abundance of either *ILT2* or *PD-L1*. Asterisks indicate significant correlations (unadjusted Pearson correlation two-sided *P* < 0.05). ILT2 is also known as LILRB1 and PD-L1 as CD274. CD, cluster of differentiation; cpm, counts per million reads mapped; DC, dendritic cell; ILT2, immunoglobulin-like transcript 2; LILRB, leukocyte immunoglobulin-like receptor subfamily B; NK, natural killer; PD-L1, programmed death-ligand 1; RNA-seq, ribonucleic acid sequencing.

To determine how the IMMUNOME observations from pre-treatment peripheral blood translate to the tumor microenvironment, we analyzed gene expression at the single-cell level in tumor tissue. Single-cell RNA-sequencing (scRNA-seq) data from 15 surgical tumor samples were analyzed in an exploratory fashion to assess cells expressing the markers of interest. In tumor tissue, NKG2A and KIRs, including KIR2DL1, were predominantly expressed on NK cells (Fig. 4 and Extended Data Fig. 8); these receptors were more highly expressed on a greater percentage of NK cells in patients with lesser pathological regression (Fig. 4). ILT2 in tumor tissue, by contrast, was expressed to only a small extent on NK cells and was instead predominantly expressed on dendritic cells (DCs), macrophages and monocytes. PD-L1 was mainly expressed on DCs and common myeloid progenitor cells and to a lesser extent in tumor tissue (Fig. 4 and Extended Data Fig. 9).

**Extended Data Fig. 10:**
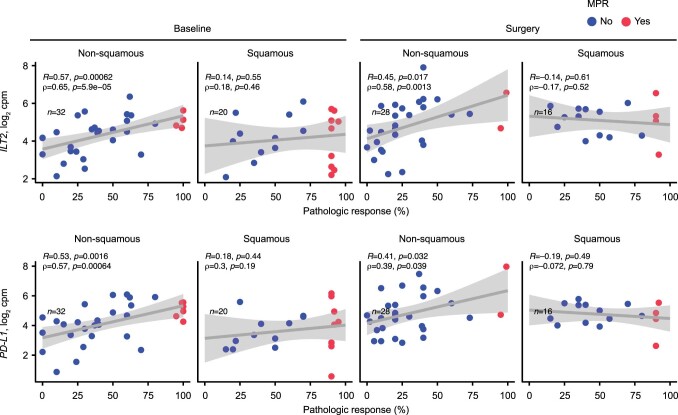
Association of *ILT2* and *PD-L1* transcripts with pathologic response at baseline and at surgery by tumor histology. Expression values for each transcript are presented as log_2_ (cpm + 1). Two-sided *P-*values for Pearson (R) and Spearman (ρ [Rho]) correlation are shown. The grey band represents the 95% confidence interval for the linear regression line. No adjustment was made for multiplicity. Of the 54 patients at baseline with RNA-seq data, 52 also had pathologic response data available. ILT2 is also known as LILRB1 and PD-L1 as CD274. CD, cluster of differentiation; cpm, counts per million reads mapped; ILT2, immunoglobulin-like transcript 2; LILR1, leukocyte immunoglobulin-like receptor 1; MPR, major pathologic response; PD-L1, programmed death-ligand 1; RNA-seq, ribonucleic acid sequencing.

Bulk RNA-seq data were available for 54 patients at baseline and 44 patients at surgery. In exploratory analyses, *ILT2* and *PD-L1* transcripts in the tumor sample assessed via bulk RNA-seq were significantly associated with pathological response in non-squamous tumors at baseline and surgery (Extended Data Fig. 10).

## Discussion

This phase II LCMC3 study of neoadjuvant atezolizumab, the largest study of preoperative checkpoint inhibitor monotherapy in early-stage NSCLC to date, met its primary end point with an MPR rate of 20% (6% pCR) in primary tumors from patients with resectable stage IB–IIIB NSCLC. Neoadjuvant atezolizumab was well tolerated, with a low incidence of treatment-related grade ≥3 AEs. There was only one treatment-related death (immune-mediated pneumonitis), the onset of which occurred 1 month after surgery; the patient died despite optimal medical management. The composite perioperative mortality rate in LCMC3 was equivalent to that of neoadjuvant chemotherapy and of surgery without chemotherapy^5^. Overall, the safety profile was consistent with that observed in advanced disease^6–8^. Biomarkers predictive of drug toxicity remain an unmet need. Despite a high-risk population, including approximately half of patients with clinical stage III disease, 88% of patients had planned surgery. Median DFS and OS were not reached, with an encouraging 3-year OS rate of 80%.

MPR was selected as the primary end point for this study to provide reasonable comparison to historical studies of neoadjuvant chemotherapy, in which MPR rates of 15–22% have been reported, rather than single-digit pCR rates^9,10^. These response rates have recently been substantiated in the randomized chemotherapy arm of CheckMate 816, reporting an MPR rate of 9% and pCR rate of 2%^11^. Small studies of neoadjuvant nivolumab monotherapy have demonstrated MPR rates of 22–45%^2,3^. Although MPR rates of 57–83% following neoadjuvant chemoimmunotherapy have recently been reported^12,13^, the addition of chemotherapy may confound the interpretation of immune predictors. Moreover, the results of these small studies have large confidence intervals. Larger studies, such as LCMC3 and CheckMate 816, provide more accurate MPR rates following neoadjuvant treatment, with an MPR rate in the tumor post-atezolizumab monotherapy of 20% and in the tumor and lymph nodes post-nivolumab plus chemotherapy of 37%^11^. The ability to select patients for the most effective systemic regimen—single-agent immunotherapy, chemotherapy or chemoimmunotherapy—remains a major unmet clinical need. The phase III IMpower030 study of neoadjuvant atezolizumab and chemotherapy is ongoing.

LCMC3 is also the largest analysis of pre- and post-treatment samples in patients with NSCLC treated with single-agent immunotherapy, enabling the robust study of predictive biomarkers. Consistent with findings in the metastatic setting, MPR was associated with high PD-L1 TPS, but as PD-L1 status was unknown for 19% of patients, this outcome should be interpreted with caution. We show in a rigorous training–testing analysis that multi-lineage immunophenotyping of a pre-treatment peripheral blood sample provides information that may predict the probability of pathological response. Consistent with previous studies of patients with NSCLC treated with PD-(L)1 blockade, we observed significant expansion of peripheral blood-activated CD8^+^ T cells in patients with tumors demonstrating MPR^14–16^. Unexpectedly, we also found significant associations between NK and NK-like T cell markers in both the peripheral blood and tumor tissue and response. Specifically, high pre-treatment levels of NK-like T cells and NK cells, including subsets expressing ILT2^+^NKG2A^+^, in the peripheral blood were significantly associated with lack of response, a result verified in two test cohorts. Expression of ILT2, which binds to human leukocyte antigen (HLA)-G, on NK cells and invariant NK T cells contributes to tumor tolerance by reducing the proliferative and cytotoxic activities of these cells^17–20^. NKG2A is expressed by immature NK cells^21,22^ and binds to HLA-E^23,24^, the expression of which is increased in various solid tumors^25–27^. NKG2A can suppress the proliferation of NK cells and NK cell-mediated cytotoxicity^22,24,26,27^. Antibodies directed against both ILT2/HLA-G and NKG2A/HLA-E are being investigated as therapeutic agents.

In our scRNA-seq data, NK cells in tumor tissue showed higher expression of NKG2A and KIRs, including KIR2DL1, on a larger percentage of NK cells in patients with less pathological regression, suggesting that these subsets impair responses to anti-PD-L1. Differential expression of KIR receptors on tumor-infiltrating lymphocytes of patients with NSCLC treated with neoadjuvant nivolumab has been shown to be associated with MPR^28^. ILT2 in tumor tissue was instead predominantly expressed on DCs, macrophages and monocytes and was positively associated with MPR. The two non-T/non-NK cell subsets that we found to positively associate with MPR in the peripheral blood immunophenotyping analysis express *ILT2* and the NKG2A-related molecule NKG2D and therefore are possibly of myeloid lineage^29^. *ILT2* and *PD-L1* transcripts in tumor samples assessed via bulk RNA-seq were significantly associated with pathological regression in non-squamous tumors at baseline and surgery. Notably, we found PD-L1 to be mainly expressed on DCs and common myeloid progenitor cells and to a lesser extent in tumor tissue. Recent literature suggests that ILT2 and PD-L1 are upregulated on DCs following antigen stimulation and may protect activated DCs from CD8^+^ T cell attack^30,31^. Thus, our data suggest that not only the adaptive immune system but also the innate immune system in the circulation and tumor tissue play a role in mediating antitumor immune responses to neoadjuvant anti-PD-L1 therapy.

We recognize that interpretation of our predictive biomarker analyses is limited because of the single-arm design of this study. Additional studies are needed to validate the significantly associated cell subsets and further establish the role of peripheral NK cells and NK-like T cells, as well as DCs in tumor tissue, in antitumor responses to immune-checkpoint inhibitors in NSCLC. Following external validation, the value of these cell subsets in predicting outcomes in the clinical practice setting must be determined. Each IMMUNOME panel subset was limited to ten markers; therefore, cells with markers of interest may overlap between subsets (Supplementary Tables 5, 7 and 9). We also acknowledge as a limitation that MPR (as defined by Pataer)^10^ was assessed only on the primary tumor; data on the lymph nodes were exploratory and will be presented elsewhere.

In conclusion, neoadjuvant treatment with single-agent atezolizumab yielded a 20% MPR rate in patients with stage IB–III NSCLC, with no new safety signals and encouraging survival. These data confirm that anti-PD-L1 monotherapy is effective in a subset of patients and begins to address two major unmet needs: understanding which biomarkers are predictive of immunotherapy response and identifying patients who may not need chemotherapy. Our biomarker analyses showed that pre-treatment peripheral blood immune cell profiles may predict MPR in atezolizumab-treated patients with resectable NSCLC. Although confirmatory and functional studies are needed, the insights from this analysis also suggest an important role for the innate immune system in the context of PD-(L)1 inhibition and the potential for new treatment regimens involving agents that modulate ILT2/HLA-G and NKG2A/HLA-E.

## Methods

### Study design

LCMC3 was an open-label, single-arm, phase II study (NCT02927301) of atezolizumab administered to patients with NSCLC before curative-intent surgery. The study, performed at 13 sites in the United States (Supplementary Table 10), consisted of two parts: a neoadjuvant phase and an optional adjuvant phase (Extended Data Fig. 1).

Patients were scheduled to receive two doses of atezolizumab 1,200 mg intravenously, given 3 weeks apart before planned surgical resection. Dose delays, but not modifications, were permitted. Tumors were staged at screening by computed tomography (CT) of the chest, positron emission tomography, brain magnetic resonance imaging and pathological confirmation of nodal involvement when appropriate. Scans were repeated before resection to assess response and confirm surgical eligibility. Tumor, lymph node (when feasible) and whole blood samples were obtained before neoadjuvant atezolizumab and at surgery.

Postoperatively, patients were permitted to receive standard-of-care adjuvant chemotherapy ± thoracic radiotherapy. Thereafter, patients with absence of radiographical progression following neoadjuvant atezolizumab and complete resection were permitted to receive adjuvant atezolizumab for up to 12 months. CT scans of the chest were acquired after surgery, then every 3–6 months thereafter for up to 2 years; additional imaging was obtained as clinically indicated.

### Patients

Participants were ≥18 years old, had pathologically documented stage IB–IIIB NSCLC as per the American Joint Committee on Cancer Staging System (8th edition)^32^ and were deemed surgically resectable and functionally operable by the treating physicians. Patients had disease that was measurable per RECIST (v.1.1)^33^ and an ECOG performance status score 0–1. The status of any actionable biomarker was not a condition of enrollment. Exclusion criteria included history of lung cancer in the preceding 3 years, previous treatment with a PD-1/PD-L1 inhibitor, major surgical procedure or severe infection in the preceding 28 d, history of autoimmune disease and history of idiopathic pulmonary fibrosis, pneumonitis, organizing pneumonia or evidence of active pneumonitis on chest CT.

### Study oversight

LCMC3 was conducted in compliance with the Declaration of Helsinki and International Conference on Harmonization Guidelines for Good Clinical Practice and was approved by the institutional review board at each participating site (Washington University School of Medicine; New York University; The Ohio State University; Karmanos Cancer Institute; Brigham and Women’s Hospital and Dana-Farber Cancer Institute; City of Hope Comprehensive Cancer Center; Moffitt Cancer Center; UCLA Community Oncology Practice; Dartmouth-Hitchcock Medical Center; University of Colorado Cancer Center; Memorial Sloan Kettering Cancer Center; Winship Cancer Institute, Emory University School of Medicine; and Yale Cancer Center). All patients provided written informed consent.

### Outcomes

Clinical data were captured electronically using Medidata Classic Rave (v.2019.2.0). Per protocol, the primary end point was MPR (≤10% viable malignant cells per local pathology assessment^34^) in the primary tumor at resection; patients whose tumors had *EGFR* or *ALK* alterations were excluded. MPR was assessed locally per study-specific pathology training and standard operating procedures^35,36^ and subsequently reviewed by a central pathology committee^35–37^. Prior analysis showed good inter-reader agreement between the local and central pathologists^38^. Secondary end points included investigator-assessed objective response rate by RECIST, pCR, pathological response by PD-L1 expression and tumor mutational burden and safety (as per the Common Terminology Criteria for Adverse Events, v.4.0). PD-L1 status was centrally determined by immunohistochemistry using the DAKO PD-L1 (22C3) assay.

Exploratory end points for patients with an MPR assessment included DFS (time from surgery to disease recurrence or death from any cause) and OS (time from first atezolizumab dose to death from any cause). Correlative analyses included paired-exome sequencing of tumor and blood DNA, peripheral blood immunophenotyping (IMMUNOME; Supplementary Table 5 and Extended Data Fig. 5) and RNA-seq and scRNA-seq of tumor samples.

### Tumor mutation status

Patients were required to provide at least two cores of pre-treatment, formalin-fixed, paraffin-embedded (FFPE) tumor tissue and at least one core of fresh frozen tissue from the primary tumor. For each patient, EGFR and ALK status were determined on the basis of local genotyping, exome sequencing on a pre-surgical sample or exome sequencing on a resection sample. ALK status was also determined by immunohistochemistry (Ventana ALK immunohistochemistry, D5F3).

### Exome sequencing

DNA and RNA were extracted from FFPE tissue using the AllPrep DNA/RNA FFPE kit (Purigen Biosystems) and DNA was extracted from blood using the QIAsymphony SP instrument (QIAGEN). For exome sequencing, the library was constructed using ≥90 ng DNA (Kapa Library Quantification Kits, Illumina), followed by hybrid capture using Nextera Rapid Capture Enrichment (Illumina), with a target of 37 Mb and sequencing on HiSeq 2500, HiSeq v.4, NovaSeq, HiSeq X, or HiSeq 4000 machines (Illumina), to generate paired-end 76-bp reads; and identification quality control check. After removing reads with low nucleotide qualities (70% of bases with quality <23), FASTQ reads were aligned to the human reference genome (Genome Reference Consortium Human Build 38 [GRCh38]) using the Genomic Short-Read Nucleotide Alignment Program (GSNAP)^39,40^ v2013-10-10 (parameters: ‘-M 2 -n 10 -B 2 -i 1 --pairmax-dna=1000 --terminal-threshold=1000 --gmap-mode=none --clip-overlap’).

Duplicate reads in the resulting BAM file were marked using PicardTools and insertions/deletions were realigned using the GATK IndelRealigner tool. Variations were called using the default options in Strelka (v.1.0.14) and Lofreq (v.2.1.2) in comparison of tumor sequence to a paired normal tissue sequence. The consequences of each mutation were determined using Ensembl Variant Effect Predictor (v.77). Tumor mutational burden was calculated as the number of Strelka mutations with Variant Effect Predictor consequences affecting protein sequence divided by the number of coding bases with ≥7-times unique coverage in the tumor sample; the final value was reported as the number of mutations per megabase.

### IMMUNOME

To identify immune cell subsets (features) significantly associated with MPR, we performed ten-color, 60-marker IMMUNOME flow cytometry (Navios Cytometer and Analysis Software v.2.1, Beckman Coulter Life Sciences; Supplementary Table 5 and Extended Data Fig. 5) on pre-treatment peripheral blood from patients without known *EGFR* or *ALK* alterations whose samples were processed within 72 h. IMMUNOME was performed in a Clinical Laboratory Improvement Amendments-certified clinical flow laboratory, using a panel previously validated for antibody stability. Patient samples were divided into two groups: those with an MPR assessment (*n* = 111), who were further divided into a training set (*n* = 57) to develop a predictive model and test set 1 (*n* = 54) and those for whom no MPR assessment was available because of PD (*n* = 9; test set 2). MPR was not assessed in the nine patients in test set 2 because of inoperability.

Through all possible combinations of ten cell surface markers in each of the 14 test tubes (Supplementary Table 5), 6,593 surface marker combinations were identified in the training set. Marker combinations detected in <50% of patients were excluded, leaving approximately 1,800 markers. An information divergence-based algorithm was then used to identify the marker combinations most different between extreme responders and non-responders in the training set. The algorithm used to score features was informed by normalized immunomes, consisting of 13 samples from patients with pathological regression (≤88% viable tumor cells) and 13 samples from patients with pathological progression (≥20% viable tumor cells); all 26 samples derived from patients in the training set. Briefly, this algorithm randomly subsampled 30 features (percentage abundance of cells with a specific marker combination) 1 million times. The importance of each feature in the subset was scored using the I-index^41^. Cells with impossible marker combinations (for example, both CD3^+^ and CD19^+^) were excluded, and only those with an I-index > 0 were retained, yielding 188 immunophenotypes.

The 188 immune cell subsets selected for inclusion in the correlative analyses were then divided into two groups: ‘non-prevalent’ and ‘prevalent’. Prevalent features were defined as those present in ≥85% of the training samples. To build a multiparametric model on the training set and to validate its performance on the testing set, non-prevalent and prevalent features were further filtered using chi-squared statistics and *t*-tests (or nonparametric alternatives), respectively, resulting in the inclusion of 17 ‘non-prevalent’ and 10 ‘prevalent’ immunophenotypes in the initial model (GAM–LASSO)^42^. The robustness of the immune cell subset selections was cross-validated and significance was tested in the training set. ROC curves were used to test the discriminative power of the selected models. To avoid overfitting, only the best-performing ‘prevalent’ feature was included in the additive part of the final model; the best-performing model with this constraint included 1 ‘prevalent’ and 13 ‘non-prevalent’ immunophenotypes. To evaluate its ability to predict the probability of MPR, we applied this model to test set 1 and then to test set 2. The probability of MPR between the different sets was compared using *t*-tests.

The ten-color IMMUNOME development and validation was performed by the clinical flow cytometry laboratory at the Ohio State University Medical Center.

### Bulk RNA-seq

RNA (extracted as above) was quantified using the Quant-iT RiboGreen RNA Assay kit (Thermo Fisher Scientific). RNA Quality Score (RQS) was determined using the LabChip GX Touch nucleic acid analyzer (PerkinElmer). RNA samples with RQS > 5.5 were analyzed by standard polyA^+^ capture RNA-seq using the TruSeq Stranded mRNA Library Prep kit (Illumina); samples with RQS < 5.5 were sequenced using Transcriptome Capture v.1 (Broad Institute). Complementary DNA libraries were sequenced on HiSeq 2500, HiSeq v.4, NovaSeq, HiSeq X or HiSeq 4000 machines (Illumina), which generated paired-end 101-bp reads. RNA-seq reads were aligned to GRCh38 using GSNAP^39,40^ v.2013-10-10, which permitted a maximum of two mismatches per 75-base sequence (parameters were: ‘-M 2 -n 10 -B 2 -i 1 -N 1 -w 200,000 -E 1–pairmax-rna = 200,000 –clip-overlap).

To quantify gene expression levels, we calculated the number of reads mapped to exons in each RefSeq gene using the functionality provided by the R/Bioconductor package GenomicAlignments. Raw counts were normalized to cpm. Batch effects due to sequencing date and library preparation kit were removed using the R package limma^43^. After discarding genes not present at ≥0.5 c.p.m. in ≥10% of samples (due to low abundance), 17,729 genes remained for analysis. xCell (v.1.3) was used to identify enriched cell subsets in pre- and post-treatment samples on the basis of the relative abundance of their transcriptomes^44^.

### scRNA-seq

Fresh tumor samples were collected from patients enrolled after 4 January 2019. In total, 15 surgical samples from six patients with NSCLC and nine with squamous NSCLC were analyzed via scRNA-seq. Briefly, fresh tumor and matched-normal samples were dissociated into single-cell suspensions using the Human Tumor Dissociation kit (Miltenyi Biotec) and gentleMACS Dissociator (Miltenyi Biotec). Erythrocytes were removed using the Red Blood Cell Lysis Solution kit (Miltenyi Biotec). After washing in cold PBS, sample viability (>70%) was confirmed using Trypan blue staining. Viable samples were then loaded onto a Chromium Controller (10x Genomics). Droplet emulsions were immediately recovered for reverse transcription via a Bio-Rad thermocycler. Single-cell expression libraries were constructed using the Chromium Single Cell 5′ Feature Barcode Library kit (v.1) (10x Genomics), the quality of which was assessed using the BioAnalyzer High Sensitivity DNA kit (Agilent). The resulting libraries were then sequenced using NextSeq 500 (Illumina).

Raw sequencing data were aligned to the GRCh38 reference genome using Cell Ranger pipeline (10x Genomics) to generate gene-cell count matrices. Data normalization and integration were performed using the Seurat R package (v.4.0.2). Cells were filtered from the downstream analysis using the following criteria: <200 or >6,000 genes detected and >0.1 fraction of mitochondrial genes. The integrated Seurat object was further scaled by regressing out unique molecular identifier count and the fraction of mitochondrial genes. The optimal principal component for dimensionality reduction was determined empirically for each analysis by the drop-off in principal component variance.

### Statistical analyses

An MPR rate of ≥15% was selected as evidence of clinical efficacy based on a previous study^45^. To provide 95% power to detect a 10% difference (null hypothesis 5%) at a one-sided significance level of 0.05, we targeted 180 patients for enrollment. Tumors from patients with incomplete surgical resection were considered to not have MPR. Patients who did not undergo surgery following neoadjuvant atezolizumab were not evaluable for MPR. All atezolizumab-treated patients with NSCLC were included in the safety population. The Kaplan–Meier method was used for the survival analyses, which were performed in the subset of patients in the efficacy population with R0 resection. The data cutoff date was 15 October 2021. Statistical analyses were performed using SAS Proprietary Software (v.9.4; SAS Institute), R v.4.1.0, ggplot2_3.3.5, ggpubr_0.4.0 and R v.4.0.5 (2021-03-31).

### Reporting summary

Further information on research design is available in the Nature Research Reporting Summary linked to this article.

## Online content

Any methods, additional references, Nature Research reporting summaries, source data, extended data, supplementary information, acknowledgements, peer review information; details of author contributions and competing interests; and statements of data and code availability are available at 10.1038/s41591-022-01962-5.

### Supplementary information

Supplementary Information Supplementary Tables 1–10 and Supplementary Figs. 1–10

Reporting Summary

## Data Availability

Complete de-identified patient data will be available indefinitely within 2 years after the last patient’s last survival follow-up visit. Qualified researchers may request access to individual patient-level clinical data through Vivli (data request platform used at the time of this writing) at https://vivli.org/ourmember/roche/. For up-to-date details on Roche’s Global Policy on the Sharing of Clinical Information and how to request access to related clinical study documents, see https://go.roche.com/data_sharing. Anonymized records for individual patients across more than one data source external to Roche cannot, and should not, be linked due to a potential increase in the risk of patient re-identification. Requests for the exploratory biomarker data underlying this publication should be directed to LCMC3_Core_Study_Team@gene.com for consideration. Data from the Genome Reference Consortium Human Build 38 can be accessed at www.ncbi.nlm.nih.gov/assembly/GCF_000001405.26/.

## Code availability

The custom computer code used to generate the IMMUNOME results reported in this paper can be accessed at 10.5281/zenodo.6811671.

## References

[1] Wakelee HA (2021). IMpower010: Primary results of a phase III global study of atezolizumab versus best supportive care after adjuvant chemotherapy in resected stage IB-IIIA non-small cell lung cancer (NSCLC). J. Clin. Oncol..

[2] Forde PM (2018). Neoadjuvant PD-1 blockade in resectable lung cancer. N. Engl. J. Med..

[3] Cascone T (2021). Neoadjuvant nivolumab or nivolumab plus ipilimumab in operable non-small cell lung cancer: the phase 2 randomized NEOSTAR trial. Nat. Med..

[4] Herbst RS (2014). Predictive correlates of response to the anti-PD-L1 antibody MPDL3280A in cancer patients. Nature.

[5] Scagliotti GV (2012). Randomized phase III study of surgery alone or surgery plus preoperative cisplatin and gemcitabine in stages IB to IIIA non-small-cell lung cancer. J. Clin. Oncol..

[6] West H (2019). Atezolizumab in combination with carboplatin plus nab-paclitaxel chemotherapy compared with chemotherapy alone as first-line treatment for metastatic non-squamous non-small-cell lung cancer (IMpower130): a multicentre, randomised, open-label, phase 3 trial. Lancet Oncol..

[7] Socinski MA (2018). Atezolizumab for first-line treatment of metastatic nonsquamous NSCLC. N. Engl. J. Med..

[8] Rittmeyer A (2017). Atezolizumab versus docetaxel in patients with previously treated non-small-cell lung cancer (OAK): a phase 3, open-label, multicentre randomised controlled trial. Lancet.

[9] Chaft JE (2016). Adaptive neoadjuvant chemotherapy guided by (18)F-FDG PET in resectable non-small cell lung cancers: the NEOSCAN trial. J. Thorac. Oncol..

[10] Pataer A (2012). Histopathologic response criteria predict survival of patients with resected lung cancer after neoadjuvant chemotherapy. J. Thorac. Oncol..

[11] Forde PM (2022). Neoadjuvant nivolumab plus chemotherapy in resectable lung cancer. N. Engl. J. Med..

[12] Shu CA (2020). Neoadjuvant atezolizumab and chemotherapy in patients with resectable non-small-cell lung cancer: an open-label, multicentre, single-arm, phase 2 trial. Lancet Oncol..

[13] Provencio M (2020). Neoadjuvant chemotherapy and nivolumab in resectable non-small-cell lung cancer (NADIM): an open-label, multicentre, single-arm, phase 2 trial. Lancet Oncol..

[14] Fehlings M (2019). Late-differentiated effector neoantigen-specific CD8^+^ T cells are enriched in peripheral blood of non-small cell lung carcinoma patients responding to atezolizumab treatment. J. Immunother. Cancer.

[15] Rizvi NA (2015). Cancer immunology. Mutational landscape determines sensitivity to PD-1 blockade in non-small cell lung cancer. Science.

[16] Anagnostou V (2019). Dynamics of tumor and immune responses during immune checkpoint blockade in non-small cell lung cancer. Cancer Res..

[17] Liu L, Wang L, Zhao L, He C, Wang G (2020). The role of HLA-G in tumor escape: manipulating the phenotype and function of immune cells. Front. Oncol..

[18] Khan M, Arooj S, Wang H (2020). NK cell-based immune checkpoint inhibition. Front. Immunol..

[19] Villa-Álvarez M (2018). Ig-like transcript 2 (ILT2) blockade and lenalidomide restore NK cell function in chronic lymphocytic leukemia. Front. Immunol..

[20] Wu CL (2021). Inhibition of iNKT cells by the HLA-G-ILT2 checkpoint and poor stimulation by HLA-G-expressing tolerogenic DC. Front. Immunol..

[21] Voss SD, Daley J, Ritz J, Robertson MJ (1998). Participation of the CD94 receptor complex in costimulation of human natural killer cells. J. Immunol..

[22] Nielsen N, Ødum N, Ursø B, Lanier LL, Spee P (2012). Cytotoxicity of CD56(bright) NK cells towards autologous activated CD4^+^ T cells is mediated through NKG2D, LFA-1 and TRAIL and dampened via CD94/NKG2A. PLoS ONE.

[23] Lee N (1998). HLA-E is a major ligand for the natural killer inhibitory receptor CD94/NKG2A. Proc. Natl Acad. Sci. USA.

[24] Braud VM (1998). HLA-E binds to natural killer cell receptors CD94/NKG2A, B and C. Nature.

[25] van Hall T (2019). Monalizumab: inhibiting the novel immune checkpoint NKG2A. J. Immunother. Cancer.

[26] Kamiya T, Seow SV, Wong D, Robinson M, Campana D (2019). Blocking expression of inhibitory receptor NKG2A overcomes tumor resistance to NK cells. J. Clin. Invest..

[27] Caushi JX (2021). Transcriptional programs of neoantigen-specific TIL in anti-PD-1-treated lung cancer. Nature.

[28] André P (2018). Anti-NKG2A mAb is a checkpoint inhibitor that promotes anti-tumor immunity by unleashing both T and NK cells. Cell.

[29] Borst L, van der Burg SH, van Hall T (2020). The NKG2A–HLA-E axis as a novel checkpoint in the tumor microenvironment. Clin. Cancer Res..

[30] Carenza C (2019). Costimulatory molecules and immune checkpoints are differentially expressed on different subsets of dendritic cells. Front. Immunol..

[31] Peng Q (2020). PD-L1 on dendritic cells attenuates T cell activation and regulates response to immune checkpoint blockade. Nat. Commun..

[32] Brierley, J., Gospodarowicz, M. K. & Wittekind, C. *TNM Classification of Malignant Tumours* 8th edn (John Wiley & Sons, 2017).

[33] Eisenhauer EA (2009). New response evaluation criteria in solid tumours: revised RECIST guideline (version 1.1). Eur. J. Cancer.

[34] Pataer A (2021). Evaluation of pathologic response in lymph nodes of patients with lung cancer receiving neoadjuvant chemotherapy. J. Thorac. Oncol..

[35] Pataer A (2012). Histopathologic response criteria predict survival of patients with resected lung cancer after neoadjuvant chemotherapy. J. Thorac. Oncol..

[36] Hellmann MD (2014). Pathological response after neoadjuvant chemotherapy in resectable non-small-cell lung cancers: proposal for the use of major pathological response as a surrogate endpoint. Lancet Oncol..

[37] Travis WD (2020). IASLC multidisciplinary recommendations for pathologic assessment of lung cancer resection specimens after neoadjuvant therapy. J. Thorac. Oncol..

[38] Dacic S (2021). Artificial intelligence (AI)-powered pathologic response (PathR) assessment of resection specimens after neoadjuvant atezolizumab in patients with non-small cell lung cancer: results from the LCMC3 study. J Clin. Oncol..

[39] Wu TD, Nacu S (2010). Fast and SNP-tolerant detection of complex variants and splicing in short reads. Bioinformatics.

[40] Wu TD (2016). GMAP and GSNAP for genomic sequence alignment: enhancements to speed, accuracy, and functionality. Methods Mol. Biol..

[41] Rempala GA, Seweryn M (2013). Methods for diversity and overlap analysis in T-cell receptor populations. J. Math. Biol..

[42] The Comprehensive R Archive Network. Plsmselect: linear and smooth predictor modelling with penalisation and variable selection. https://CRAN.R-project.org/package=plsmselect (2021).

[43] Ritchie ME (2015). limma powers differential expression analyses for RNA-sequencing and microarray studies. Nucleic Acids Res..

[44] Aran D, Hu Z, Butte AJ (2017). xCell: digitally portraying the tissue cellular heterogeneity landscape. Genome Biol..

[45] Chaft JE (2016). Adaptive neoadjuvant chemotherapy guided by (18)F-FDG PET in resectable non-small cell lung cancers: the NEOSCAN trial. J. Thorac. Oncol..

