# Author Correction: Neoadjuvant atezolizumab for resectable non-small cell lung cancer: an open-label, single-arm phase II trial

**DOI:** 10.1038/s41591-023-02627-7

**Published:** 2023-10-10

**Authors:** Jamie E. Chaft, Filiz Oezkan, Mark G. Kris, Paul A. Bunn, Ignacio I. Wistuba, David J. Kwiatkowski, Dwight H. Owen, Yan Tang, Bruce E. Johnson, Jay M. Lee, Gerard Lozanski, Maciej Pietrzak, Michal Seweryn, Woo Yul Byun, Katja Schulze, Alan Nicholas, Ann Johnson, Jessica Grindheim, Stephanie Hilz, David S. Shames, Chris Rivard, Eric Toloza, Eric B. Haura, Ciaran J. McNamee, G. Alexander Patterson, Saiama N. Waqar, Valerie W. Rusch, David P. Carbone, LCMC study investigators, Saiama N. Waqar, Elaine Shum, Misako Nagasaka, Marianna Koczywas, Edward B. Garon, David J. Finley, David R. Camidge, Jennifer W. Carlisle, Justin D. Blasberg

**Affiliations:** 1 Memorial Sloan Kettering Cancer Center, New York, NY USA; 2 Weill Cornell Medical College, New York, NY USA; 3 The Ohio State University Comprehensive Cancer Center, Columbus, OH USA; 4 University Medicine Essen, Ruhrlandklinik, Department of Interventional Pulmonology, University Duisburg-Essen, Essen, Germany; 5 German Cancer Research Center (DKFZ), A420, Heidelberg, Germany; 6 Fifth Medical Department, Section of Pulmonology, Faculty of the University of Heidelberg, University Medicine Mannheim, Mannheim, Germany; 7 University of Colorado School of Medicine, Aurora, CO USA; 8 The University of Texas MD Anderson Cancer Center, Houston, TX USA; 9 Dana-Farber Cancer Institute, Boston, MA USA; 10 Brigham and Women’s Hospital, Boston, MA USA; 11 The Ohio State University Wexner Medical Center, Columbus, OH USA; 12 David Geffen School of Medicine at UCLA, Los Angeles, CA USA; 13 Biobank Lab, Department of Molecular Biophysics, University of Lodz, Lodz, Poland; 14 Centre for Data Analysis, Modeling and Computational Sciences, University of Lodz, Lodz, Poland; 15 Genentech, Inc., South San Francisco, CA USA; 16 Moffitt Cancer Center and Research Institute, Tampa, FL USA; 17 Washington University School of Medicine, St. Louis, MO USA; 18 Pelotonia Institute for Immuno-Oncology, Columbus, OH USA; 19 Alvin J. Siteman Cancer Center, St. Louis, MO USA; 20 New York University Langone Health, New York, NY USA; 21 Perlmutter Comprehensive Cancer Center, New York University, New York, NY USA; 22 Karmanos Cancer Institute, Detroit, MI USA; 23 City of Hope Comprehensive Cancer Center, Duarte, CA USA; 24 Dartmouth-Hitchcock Medical Center, Lebanon, NH USA; 25 University of Colorado Cancer Center, Aurora, CO USA; 26 Winship Cancer Institute, Emory University School of Medicine, Atlanta, GA USA; 27 Yale University School of Medicine, New Haven, CT USA

**Keywords:** Non-small-cell lung cancer, Phase II trials

Correction to: *Nature Medicine* 10.1038/s41591-022-01962-5. Published online 12 September 2022.

In the version of this article initially published, the first sentence of the Fig. 4a caption (now reading “The expression of different NK cell surface receptors was determined by scRNA-seq”) was incorrectly preceded by the text “Association between gene expression in NK cells and the percentage of viable tumor cells,” which has now been removed from the HTML and PDF versions of the article.

