# Supplementary material for: Neoadjuvant atezolizumab for resectable non-small cell lung cancer: an open-label, single-arm phase II trial
Source: Nat Med. 2022 Sep 12;28(10):2155–61. doi: 10.1038/s41591-022-01962-5 (PMC9556329; doi:10.1038/s41591-022-01962-5)
Supplement: Supplementary file 1 — Supplementary Tables 1–10 and Supplementary Figs. 1–10 [file 41591_2022_1962_MOESM1_ESM.pdf]

nature portfolio

https://doi.org/10.1038/s41591-022-01962-5

Supplementary information

Neoadjuvant atezolizumab for resectable non-small cell lung cancer: an open-label, single-arm phase II trial

In the format provided by the authors and unedited

Supplemental Table 1. Baseline demographics and disease characteristics.

	Patients (*N*=181)	Did not undergo surgery (*n*=22)	No MPR or unknown MPR status (*n*=130)	MPR documented (*n*=29)
Median age, years (range)	65.0 (37–83)	68.0 (46–81)	65.0 (37–82)	67.0 (39–83)
Female, *n* (%)	93 (51)	14 (64)	57 (44)	22 (76)
Race, *n* (%)				
White	145 (81)	16 (73)	105 (82)	24 (83)
Black/African American	13 (7)	3 (14)	7 (5)	3 (10)
Asian	9 (5)	1 (5)	8 (6)	0
Unknown	12 (7)	2 (9)	8 (6)	2 (7)
ECOG PS, *n* (%)				
0	104 (57)	13 (59)	77 (59)	14 (48)
1	77 (43)	9 (41)	53 (41)	15 (52)
Clinical stage at initial diagnosis, *n* (%)				
IB	18 (10)	0	15 (12)	3 (10)
IIA	16 (9)	2 (9)	12 (9)	2 (7)
IIB	55 (30)	8 (36)	40 (31)	7 (24)
IIIA	70 (39)	9 (41)	51 (39)	10 (34)
IIIB[a]	22 (12)	3 (14)	12 (9)	7 (24)
Histology, *n* (%)				
Non-squamous	112 (62)	12 (55)	85 (65)	15 (52)
Squamous	69 (38)	10 (45)	45 (35)	14 (48)
History of tobacco use, *n* (%)				
Never	18 (10)	2 (9)	14 (11)	2 (7)
Current	35 (19)	7 (32)	16 (12)	12 (41)
Former	128 (71)	13 (59)	100 (77)	15 (52)
Median pack-years, *n* (range)	22.75 (0–162.0)	21.25 (2.4–162.0)	20.00 (0–152.0)	26.50 (1.0–84.0)
PD-L1 TPS, *n* (%)[b]				
<1%	69 (38)	9 (41)	54 (42)	6 (21)
1%–49%	28 (15)	4 (18)	23 (18)	1 (3)
≥50%	49 (27)	3 (14)	31 (24)	15 (52)
Unknown[c]	35 (19)	6 (27)	22 (17)	7 (24)
EGFR mutation, *n* (%)[d]				
Positive	11 (6)	1 (5)	10 (8)	0
Negative	154 (85)	13 (59)	114 (88)	27 (93)
Unknown[e]	16 (9)	8 (36)	6 (5)	2 (7)
ALK rearrangement, *n* (%)[d]				
Positive	6 (3)	0	6 (5)	0
Negative	162 (90)	18 (82)	120 (92)	24 (83)
Unknown[f]	13 (7)	4 (18)	4 (3))	5 (17)

ALK, anaplastic lymphoma kinase; ECOG PS, Eastern Cooperative Oncology Group performance status; EGFR, epidermal growth factor receptor; MPR, major pathologic response; PD-L1, programmed death-ligand 1; TPS, tumor proportion score. [a]Includes T3N2 or T4 (by size criteria, not by mediastinal invasion) per the American Joint Committee on Cancer Staging System (8th edition). [b]PD-L1 status was centrally determined by immunohistochemistry using the DAKO PD-L1 (22C3) assay. [c]The large number of patients with "unknown" status was attributable to missing samples and failed testing. [d]Determined either locally or centrally from screening tissue (when adequate) or resected tumor tissue. [e]EGFR status was unknown in 16 patients (non-squamous, *n*=5; squamous, *n*=11). [f]ALK rearrangement status was unknown in 13 patients (non-squamous, *n*=5; squamous, *n*=8).

Supplemental Table 2. Characteristics of patients with pathologic complete response.

	Gender	Age, years	Tobacco history/ pack-years	Histology	Tumor stage	Baseline PD-L1 TPS, %	ALK status	EGFR status	TMB score, mutations/Mb	DFS, years
**Patient 1**	Male	72	Current/27.5	Non-squamous	IIIA	90	Unknown	Wild-type	N/A	3.4*
**Patient 2**	Male	58	Current/41	Squamous	IIIA	N/A	Unknown	Unknown	N/A	3.9*
**Patient 3**	Female	69	Previous/50	Non-squamous	IIB	0	Wild-type	Wild-type	31.04478	3.1*
**Patient 4**	Female	53	Previous/1	Non-squamous	IB	N/A	Wild-type	Wild-type	N/A	3.1*
**Patient 5**	Female	59	Current/44	Non-squamous	IIIA	N/A	Unknown	Unknown	N/A	1.4
**Patient 6**	Female	57	Previous/54	Non-squamous	IIIA	100	Wild-type	Wild-type	N/A	2.1*
**Patient 7**	Female	39	Previous/1	Non-squamous	IIIB	70	Wild-type	Wild-type	N/A	2.0*
**Patient 8**	Female	65	Current/15	Non-squamous	IIIA	90	Unknown	Wild-type	N/A	0.8

ALK, anaplastic lymphoma kinase; DFS, disease-free survival; EGFR, epidermal growth factor receptor; N/A, not available; PD-L1, programmed death-ligand 1; TMB, tumor mutational burden; TPS, tumor proportion score.

*Censored patients (ie, those without disease recurrence or death) at the time of data cutoff.

Supplemental Table 3. Subgroup analysis of MPR rates and odds of MPR (*n*=137[a]).

	MPR rate (*n*/*N*)	Odds Ratio, *P* value[b]
Pre-surgical response[c]		
PR	40.0 (4/10)	PR vs. SD: 2.6<br>*P*=0.147
SD	20.3 (2/123)	
Gender		
Female	32.4 (22/68)	Female vs. male: 4.2<br>*P*=0.002
Male	10.1 (7/69)	
Nodal stage		
N0	12.5 (7/56)	N1 vs. N0: 3.1, *P*= 0.033<br>N2 vs. N0: 2.3, *P*=0.119
N1	30.6 (11/36)	
N2	24.4 (11/45)	
Histology		
Squamous	27.4 (14/51)	Squamous vs. non-squamous: 1.8 *P*=0.166
Non-squamous	17.4 (15/86)	

MPR, major pathologic response; PR, partial response; RECIST, Response Evaluation Criteria in Solid Tumors; SD, stable disease.
[a]Six patients in the primary efficacy population had incomplete/no resection (missing MPR). [b]Two-sided *P* values are from chi-square test. [c]Per RECIST v1.1; patients do not sum to 137 because of 4 with progressive disease or missing assessments.

Supplemental Table 4. Safety during the neoadjuvant phase.

	Patients (*N*=181)		
	**Any grade, *n* (%)**	**Grade 3–4, *n* (%)**	**Grade 5, *n* (%)**
Any AE[a]	175 (97)	65 (36)	3 (2)[c]
Fatigue	71 (39)	2(1)	0
Procedural pain	53 (29)	9 (5)	0
Dyspnea	38 (21)	5 (3)	0
Nausea	37 (20)	0	0
Constipation	36 (20)	0	0
Decreased appetite	29 (16)	0	0
Cough	29 (16)	0	0
Pyrexia	29 (16)	2 (1)	0
Headache	26 (14)	0	0
Diarrhea	24 (13)	5 (3)	0
Pruritus	19 (10)	0	0
Anemia	19 (10)	2 (1)	0
Treatment-related AE[a]	110 (61)	19 (10)[b]	1 (<1%)[c]
Fatigue	36 (20)	1 (<1)	0
Immune-mediated AE	75 (41)	17 (9)	1 (<1)[c]
Treatment-related	61 (34)	15 (8)	1 (<1)[c]
AE leading to treatment discontinuation	9 (5)	2 (1)	0

AE, adverse event.
[a]Preferred terms reported in ≥10% of patients are presented. [b]The following treatment-related grade ≥3 AEs were reported in >1 patient: pneumonitis (*n*=4), pneumonia (*n*=3), colitis (*n*=2), empyema (*n*=2), and respiratory failure (*n*=2). [c]Three deaths were reported within 90 days of neoadjuvant atezolizumab: sudden death not otherwise specified, death due to disease progression, and pneumonitis. Only pneumonitis was considered related to study treatment.

Supplemental Table 5. Cell surface markers interrogated via 10-color, 60-marker IMMUNOME.

Tube number	Tube name	Cell surface marker									
1	Lymphosum	γ/δ	α/β	CD19	CD56	CD16	CD13/14	CD4	CD3	CD8	CD45
2	Activation	HLA-DR	CD69	CD19	CD56	CD16	CD134	CD4	CD3	CD8	CD45
3	Very late activation	CD107a/b	PD-L1	CD14	CD13	CD63	CD49a	CD4	CD3	CD8	CD45
4	B-cell activation	CD80	CD154	CD56/16	CD86	CD69	CD40	CD25	CD3	CD19	CD45
5	Naïve/memory cells	CD62L	CD27	CD56/16	CD45RO	CCR7	CD45RA	CD4	CD3	CD8	CD45
6	Regulatory T cells	CD54	CD152	CD25	CD39	CD127	CD11A	CD4	CD3	CD8	CD45
7	NK cells 1	CD94	NKG2D	CD3	CD56	CD117	NKG2A	CD127	CD161	CD16	CD45
8	NK cells 2	CD16	CD336	CD3	CD244	CD335	NKG2D	CD56	CD161	CD337	CD45
9	NK cells 3	CD107a/b	CD159c	CD3	KIR3DL1	KIR2DL2	NKp80	CD56	KIR2DL1	CD16	CD45
10	NK cells 4	CD63	ILT2	CD3	KIR3DL1	KIR2DL2	NKG2A	CD56	KIR2DL1	CD16	CD45
11	NK cells 5	HLA-DR	CD69	CD3	KIR3DL1	KIR2DL2	NKG2A	CD56	KIR2DL1	CD16	CD45
12	Myeloid cells	HLA-DR	CD124	CD14	LIN[*]	CD11b	CD66b	CD16	CD33	CD15	CD45
13	Senescent T cells	CD57	CD28	CD16	CD56	CD127	KLRG1	CD4	CD3	CD8	CD45
14	Dendritic cells	CD141	LIN[a]	HLA-DR	CD1c	CD33	CD1a	CD16	CD11B	CD15	CD45

ILT2 is also known as LILRB1, NKG2A as CD159a, NKG2D as CD314 and KLRK1, KIR2DL1 as CD158a, KIR2DL2 as CD158b, KIR3DL1 as CD158e1, PD-L1 as CD274, CD335 as NKp46, and CD337 as NKp30. α/β, α/β chains of the T-cell receptor; γ/δ, γ/δ chains of the T-cell receptor; CCR7, C-C motif chemokine receptor 7; CD, cluster of differentiation; HLA, human leukocyte antigen; IMMUNOME, peripheral blood immunophenotyping; KIR, killer cell immunoglobulin-like receptor; KLR, killer cell lectin-like receptor; LILRB1, leukocyte immunoglobulin-like receptor subfamily B1; LIN, lineage; NK, natural killer; NKG2, natural killer group protein 2; PD-L1, programmed death-ligand 1. [a]LIN included CD19, CD3, and CD56.

Supplemental Table 6. The dependence of effect size on clinical variables in the GAM–LASSO models.

Cell surface markers	Other clinical variables					
	No other clinical variable (IMMUNOME alone)	IMMUNOME + histology (non-SQ vs. SQ)	IMMUNONE + sex (female vs. male)	IMMUNONE + nodal status (N1/N2 vs. N0)	IMMUNONE + smoking (never vs. current/former)	IMMUNONE + PD-L1 expression[a]
Overall *training* AUC when peripheral blood IMMUNOME is considered in addition to:	0.987	0.987	0.994	0.987	0.989	1.000
Overall *testing* AUC when peripheral blood IMMUNOME is considered in addition to:	0.722	0.722	0.730	0.720	0.720	0.706
Coefficient (effect size) when only another clinical variable is considered (i.e., peripheral blood IMMUNOME not considered):	—	0	−0.465	0.500	0.106	0.003
NK-like T cells: CD45$^+$ CD94$^-$ NKG2D$^+$ CD3$^+$ CD56$^+$ CD117$^-$ NKG2A$^-$ CD127$^+$ CD161$^+$ CD16$^-$ (effect size)	−1.755	−1.663	0.328	−1.715	−1.729	−1.319
NK-like T cells: CD45$^+$ γ/δ$^-$ α/β$^-$ CD19$^-$ CD56$^+$ CD16$^+$ CD13/14$^-$ CD4$^-$ CD3$^+$ CD8$^-$ (effect size)	−1.323	−1.263	−1.560	−1.346	−1.308	−0.168
Degranulated myeloid cells: CD45$^+$ CD107a/b$^+$ PD-L1$^-$ CD14$^-$ CD13$^+$ CD63$^+$ CD49a$^-$ CD4$^-$ CD3$^-$ CD8$^-$ (effect size)	−1.138	−1.046	−0.600	−1.164	−1.127	0
NK-like T cells: CD45$^+$ γ/δ$^+$ α/β$^-$ CD19$^-$ CD56$^+$ CD16$^+$ CD13/14$^-$ CD4$^-$ CD3$^+$ CD8$^-$ (effect size)	−0.935	−0.923	−0.898	−0.957	−0.947	0
γ/δ T cells: CD45$^+$ γ/δ$^+$ α/β$^-$ CD19$^-$ CD56$^-$ CD16$^+$ CD13/14$^-$ CD4$^-$ CD3$^+$ CD8$^+$ (effect size)	−0.837	−0.815	−0.531	−1.067	−0.768	−0.247
NK-like T cells: CD45$^+$ γ/δ$^-$ α/β$^+$ CD19$^-$ CD56$^+$ CD16$^-$ CD13/14$^-$ CD4$^+$ CD3$^+$ CD8$^+$ (effect size)	−0.823	−0.772	−1.038	−0.586	−0.820	0
Naive CD4+/CD8+ T cells: CD45$^+$ CD62L$^-$ CD27$^+$ CD56/16$^-$ CD45RO$^-$ CCR7$^-$ CD45RA$^+$ CD4$^+$ CD3$^+$ CD8$^+$ (effect size)	−0.569	−0.510	1.208	−0.495	−0.545	0.340
NK cells: CD45$^+$ CD16$^+$ CD336$^-$ CD3$^-$ CD244$^-$ CD335$^+$ NKG2D$^-$ CD56$^-$ CD161$^-$ CD337$^+$ (effect size)	−0.497	−0.449	1.976	−0.362	−0.514	−3.619
NK cells: CD45$^+$ CD63$^-$ ILT2$^+$ CD3$^-$ KIR3DL1$^-$ KIR2DL2$^-$ NKG2A$^+$ CD56$^+$ KIR2DL1$^-$ CD16$^-$ (effect size)	−0.386	−0.353	−0.804	−0.341	−0.406	−0.182
NK-like T cells: CD45$^+$ HLA DR$^+$ CD69$^-$ CD19$^-$ CD56$^+$ CD16$^-$ CD134$^-$ CD4$^-$ CD3$^+$ CD8$^+$ (effect size)	−0.378	−0.372	−0.301	−0.392	−0.353	−0.010
NK-like T cells: CD45$^+$ CD63$^-$ ILT2$^+$ CD3$^+$ KIR3DL1$^-$ KIR2DL2$^-$ NKG2A$^+$ CD56$^+$ KIR2DL1$^-$ CD16$^-$ (effect size)	−0.324	−0.310	−1.529	−0.394	−0.323	0
Non-T/non-NK cells: CD45$^+$ CD94$^-$ NKG2D$^+$ CD3$^-$ CD56$^-$ CD117$^-$ NKG2A$^-$ CD127$^-$ CD161$^-$ CD1644$^-$ (effect size)	0.663	0.631	−1.242	0.591	0.671	0
Non-T/non-NK cells: CD45$^+$ CD63$^-$ ILT2$^+$ CD3$^-$ KIR3DL1$^-$ KIR2DL2$^+$ NKG2A$^-$ CD56$^-$ KIR2DL1$^+$ CD16$^-$ (effect size)	1.570	1.456	−0.383	1.537	1.583	0
T cells: CD45$^+$ CD94$^-$ NKG2D$^+$ CD3$^+$ CD56$^-$ CD117$^-$ NKG2A$^+$ CD127$^+$ CD161$^-$ CD16$^-$ (chi-square *P* value in GAM)	0.035	0.038	0.073	0.029	0.039	0.136

The numbers represent effect sizes, and the columns represent the different models. Shaded rows represent immune cell subsets that were more prevalent in patients with MPR. Non-shaded rows represent immune cell subsets that were more prevalent in patients with non-MPR. ILT2 is also known as LILRB1, NKG2A as CD159a, NKG2D as CD314 and KLRK1, KIR2DL1 as CD158a, KIR2DL2 as CD158b, KIR3DL1 as CD158e1, CD335 as NKp46, and CD337 as NKp30. α/β, α/β chains of the T-cell receptor; γ/δ, γ/δ chains of the T-cell receptor; AUC, area under the curve; CCR7, C-C motif chemokine receptor 7; CD, cluster of differentiation; GAM–LASSO, generalized additive model–least absolute shrinkage and selection operator; HLA, human leukocyte antigen; ILT2, immunoglobulin-like transcript 2; IMMUNOME, peripheral blood immunophenotyping; KIR, killer cell immunoglobulin-like receptor; KLR, killer cell lectin-like receptor; LILRB, leukocyte immunoglobulin-like receptor subfamily B; MPR, major pathologic response; NK, natural killer; NKG2, natural killer group protein 2; PD-L1, programmed death-ligand 1; SQ, squamous. [a]Determined as a continuous via immunohistochemistry using the DAKO PD-L1 [22C3] antibody (tumor proportion score).

Supplemental Table 7. Poisson regression between the total number of peripheral cells at baseline and MPR (training set and test set 1 merged).

Immune cell subsets <u>positively</u> associated with MPR in the GAM–LASSO model:

CD45$^+$ CD94$^-$ NKG2D$^+$ CD3$^-$ CD56$^-$ CD117$^-$ NKG2A$^-$ CD127$^-$ CD161$^-$ CD16$^-$ (non-T/non-NK cells, possible myeloid lineage)

CD45$^+$ CD63$^-$ ILT2$^+$ CD3$^-$ KIR3DL1- KIR2DL2$^+$ NKG2A$^-$ CD56$^-$ KIR2DL1$^+$ CD16$^-$ (non-T/non-NK cells, possible myeloid lineage)

	Estimate (SE)	Z	*P* value
(Intercept)	−0.06 (0.11)	−0.54	0.59
MPR	0.40 (0.20)	1.96	0.05

Immune cell subsets <u>negatively</u> associated with MPR in the GAM–LASSO model:

CD45$^+$ HLA-DR$^+$ CD69$^-$ CD19$^-$ CD56$^+$ CD16$^-$ CD134$^-$ CD4$^-$ CD3$^+$ CD8$^+$ (NK-like T cells)

CD45$^+$ γ/δ$^-$ α/β$^-$ CD19$^-$ CD56$^+$ CD16$^+$ CD13/14$^-$ CD4$^-$ CD3$^+$ CD8$^-$ (NK-like T cells)

CD45$^+$ γ/δ$^-$ α/β$^+$ CD19$^-$ CD56$^+$ CD16$^-$ CD13/14$^-$ CD4$^+$ CD3$^+$ CD8$^+$ (NK-like T cells)

CD45$^+$ γ/δ$^+$ α/β$^-$ CD19$^-$ CD56$^-$ CD16$^+$ CD13/14$^-$ CD4$^-$ CD3$^+$ CD8$^+$ (γ/δ T cells)

CD45$^+$ γ/δ$^+$ α/β$^-$ CD19$^-$ CD56$^+$ CD16$^+$ CD13/14$^-$ CD4$^-$ CD3$^+$ CD8$^-$ (γ/δ NK-like T cells)

CD45$^+$ CD94$^-$ NKG2D$^+$ CD3$^+$ CD56$^+$ CD117$^-$ NKG2A$^-$ CD127$^+$ CD161$^+$ CD16$^-$ (NK-like T cells)

CD45$^+$ CD63$^-$ ILT2$^+$ CD3$^+$ KIR3DL1$^-$ KIR2DL2$^-$ NKG2A$^+$ CD56$^+$ KIR2DL1$^-$ CD16$^-$ (NK-like T cells)

CD45$^+$ CD16$^+$ CD336$^-$ CD3$^-$ CD244$^-$ CD335$^+$ NKG2D$^-$ CD56$^-$ CD161$^-$ CD337$^+$ (NK cells)

CD45$^+$ CD63$^-$ ILT2$^+$ CD3$^-$ KIR3DL1$^-$ KIR2DL2$^-$ NKG2A$^+$ CD56$^+$ KIR2DL1$^-$ CD16$^-$ (NK cells)

CD45$^+$ CD107a/b$^+$ PD-L1$^-$ CD14$^-$ CD13$^+$ CD63$^+$ CD49a$^-$ CD4$^-$ CD3$^-$ CD8$^-$ (degranulated myeloid cells)

CD45$^+$ CD62L$^-$ CD27$^+$ CD56/16$^-$ CD45RO$^-$ CCR7$^-$ CD45RA$^+$ CD4$^+$ CD3$^+$ CD8$^+$ (naïve CD4$^+$/CD8$^+$ T cells)

	Estimate (SE)	Z	*P* value
(Intercept)	2.06 (0.04)	53.68	<0.0001
MPR	−0.24 (0.09)	−2.68	0.0073

The effect size represents the strength of the association between the grouping of all positive or negative predictors and MPR. ILT2 is also known as LILRB1, NKG2A as CD159a, NKG2D as CD314 and KLRK1, KIR2DL1 as CD158a, KIR2DL2 as CD158b, KIR3DL1 as CD158e1, CD335 as NKp46, and CD337 as NKp30. α/β, α/β chains of the T-cell receptor; γ/δ, γ/δ chains of the T-cell receptor; CCR7, C-C motif chemokine receptor 7; CD, cluster of differentiation; GAM–LASSO, generalized additive model–least absolute shrinkage and selection operator; HLA, human leukocyte antigen; ILT2, immunoglobulin-like transcript 2; KIR, killer cell immunoglobulin-like receptor; KLR, killer cell lectin-like receptor; LILRB, leukocyte immunoglobulin-like receptor subfamily B; MPR, major pathologic response; NK, natural killer; NKG2, natural killer group protein 2; PD-L1, programmed death-ligand 1; SE, standard error.

Supplemental Table 8. Clinical characteristics and their association with pathologic response in the training and test sets.

	**Training set (*n*=57)**	**Test set 1 (*n*=54)**	**Test set 2 (*n*=9)**
Median age, years (range)	65.0 (48–83)	65.0 (39–81)	68.0 (46–74)
P value[a]	0.60	0.72	—
Gender, *n* (%)			
Female	29 (51)	26 (48)	7 (78)
Male	28 (49)	28 (52)	2 (22)
P value[b]	0.12	0.007	—
Histology, *n* (%)			
Non-squamous	34 (60)	32 (60)	6 (67)
Squamous	23 (40)	22 (40)	3 (33)
P value[b]	0.27	1.00	—
History of tobacco use, *n* (%)			
Never	0	6 (11)	0
Current	13 (23)	6 (11)	3 (33)
Former	44 (77)	42 (78)	6 (67)
P value[b]	0.033	0.0009	—
Nodal stage, *n* (%)			
N0	28 (49)	19 (35)	2 (22)
N1	17 (30)	12 (21)	3 (33)
N2	12 (21)	23 (43)	4 (44)
P value[b]	0.22	0.18	—
Median PD-L1 TPS, % (range)	10 (0–100)	30 (0–100)	10 (0–90)
P- value[a]	0.087	0.0452	—

The presented *P* values are for the evaluation of a given feature of MPR. Because of the small number of patients, it was not possible to calculate *P* values in test set 2. [a]t test *P* value for the association with MPR. [b]Chi-square *P* value for the association with MPR. MPR, major pathologic response; PD-L1, programmed death-ligand 1; TPS, tumor proportion score.

Supplemental Table 9. Association between the changes in immune cell subset abundance (before and after treatment with neoadjuvant atezolizumab) and MPR.

Cell surface markers	Status in patients with MPR	Effect size
CD45^{+} CD63^{-} ILT2^{+} CD3^{-} KIR3DL1^{-} KIR2DL2^{+} NKG2A^{-} CD56^{-} KIR2DL1^{+} CD16^{-} (non-T/non-NK cells)	Expanded	7.614576
CD45^{+} CD94^{+} NKG2D^{-} CD3^{+} CD56^{+} CD117^{-} NKG2A^{+} CD127^{-} CD161^{+} CD16^{+} (NK-like T cells)	Expanded	6.922224
CD45^{+} HLA-DR^{+} CD69^{+} CD19^{-} CD56^{-} CD16^{+} CD134^{-} CD4^{-} CD3^{+} CD8^{+} (activated CD8+ T cells)	Expanded	5.267795
CD45^{+} CD107a/b^{+} CD159c^{-} CD3^{-} KIR3DL1^{-} KIR2DL2^{-} NKp80^{+} CD56^{-} KIR2DL1^{-} CD16- (activated CD56^{-}/CD16^{-} NK cells)	Expanded	1.962242
CD45^{+} CD94^{-} NKG2D^{+} CD3^{+} CD56^{-} CD117^{-} NKG2A^{+} CD127^{+} CD161^{-} CD16^{+} (T cells)	Expanded	1.584795
CD45^{+} CD94^{+} NKG2D^{+} CD3^{+} CD56^{+} CD117^{-} NKG2A^{+} CD127^{+} CD161^{-} CD16^{-} (NK-like T cells)	Contracted	-2.131951
CD45^{+} CD62L^{-} CD27^{+} CD56/16^{-} CD45RO^{+} CCR7^{+} CD45RA^{+} CD4^{+} CD3^{+} CD8^{-} (central memory CD4^{+} T cells)	Contracted	-2.239447
CD45^{+} CD1c^{+} LIN^{-} HLA-DR^{-} CD33^{-} CD16^{-} CD11b^{-} CD15^{+} (immature myeloid lineage cell)	Contracted	-3.855575
CD45^{+} CD63^{-} ILT2^{+} CD3^{-} KIR3DL1^{-} KIR2DL2^{-} NKG2A^{+} CD56^{+} KIR2DL1^{+} CD16^{+} (NK cells)	Contracted	---[a]
Chi-square value	18.39	
Two-sided *P* value	6.17e-05	

For paired samples, the difference in cell abundance was calculated, with only the direction (+1 or −1) used as a predictor in the GAM–LASSO model. The AUC of test set 1 was 0.726. LIN included CD19, CD3, and CD56. ILT2 is also known as LILRB1, NKG2A as CD159a, NKG2D as CD314 and KLRK1, KIR2DL1 as CD158a, and KIR2DL2 as CD158b. [a]The effect of the contraction of ILT2^{+} NKG2A^{+} KIR2DL1^{+} NK cells on MPR is non-linear and is reflected in the GAM part, not the linear LASSO part, of the model. The significance level for this effect equals 6.17e-05. AUC, area under the curve; CCR7, C-C motif chemokine receptor 7; CD, cluster of differentiation; GAM–LASSO, generalized additive model–least absolute shrinkage and selection operator; HLA, human leukocyte antigen; ILT2, immunoglobulin-like transcript 2; KIR, killer cell immunoglobulin-like receptor; KLR, killer cell lectin-like receptor; LILRB1, leukocyte immunoglobulin-like receptor subfamily B1; LIN, lineage; MPR, major pathologic response; NK, natural killer; NKG2, natural killer group protein 2.

Supplemental Table 10. Study sites and investigators.

Study site	Investigator
Washington University School of Medicine	Saiama N. Waqar
New York University	Elaine Shum
The Ohio State University	Dwight H. Owen
Karmanos Cancer Institute	Misako Nagasaka[a]
Brigham and Women's Hospital and Dana-Farber Cancer Institute	Ciaran McNamee
City of Hope Comprehensive Cancer Center	Marianna Koczywas
Moffitt Cancer Center	Eric B. Haura
UCLA Community Oncology Practice	Edward B. Garon
Dartmouth-Hitchcock Medical Center	David J. Finley
University of Colorado Cancer Center	David R. Camidge
Memorial Sloan-Kettering Cancer Center	Jamie E. Chaft
Winship Cancer Institute, Emory University School of Medicine	Jennifer Carlisle
Yale Cancer Center	Justin D. Blasberg

[a]Presently at University of California, Irvine, Irvine, CA, USA.
