# Supplementary material for: Neoadjuvant atezolizumab for resectable non-small cell lung cancer: an open-label, single-arm phase II trial
Source: Nat Med. 2022 Sep 12;28(10):2155–61. doi: 10.1038/s41591-022-01962-5 (PMC9556329; doi:10.1038/s41591-022-01962-5)
Supplement: Supplementary file 2 — Reporting Summary [file 41591_2022_1962_MOESM2_ESM.pdf]

nature research

Corresponding author(s): David P. Carbone

Last updated by author(s): July 1, 2022

Reporting Summary

Nature Research wishes to improve the reproducibility of the work that we publish. This form provides structure for consistency and transparency in reporting. For further information on Nature Research policies, see our Editorial Policies and the Editorial Policy Checklist.

Statistics

For all statistical analyses, confirm that the following items are present in the figure legend, table legend, main text, or Methods section.

n/a	Confirmed	
☐	☒	The exact sample size (*n*) for each experimental group/condition, given as a discrete number and unit of measurement
☐	☒	A statement on whether measurements were taken from distinct samples or whether the same sample was measured repeatedly
☐	☒	The statistical test(s) used AND whether they are one- or two-sided<br>*Only common tests should be described solely by name; describe more complex techniques in the Methods section.*
☐	☒	A description of all covariates tested
☐	☒	A description of any assumptions or corrections, such as tests of normality and adjustment for multiple comparisons
☐	☒	A full description of the statistical parameters including central tendency (e.g. means) or other basic estimates (e.g. regression coefficient) AND variation (e.g. standard deviation) or associated estimates of uncertainty (e.g. confidence intervals)
☐	☒	For null hypothesis testing, the test statistic (e.g. *F*, *t*, *r*) with confidence intervals, effect sizes, degrees of freedom and *P* value noted<br>*Give P values as exact values whenever suitable.*
☒	☐	For Bayesian analysis, information on the choice of priors and Markov chain Monte Carlo settings
☒	☐	For hierarchical and complex designs, identification of the appropriate level for tests and full reporting of outcomes
☐	☒	Estimates of effect sizes (e.g. Cohen's *d*, Pearson's *r*), indicating how they were calculated

Our web collection on statistics for biologists contains articles on many of the points above.

Software and code

Policy information about availability of computer code

Data collection	EDC (electronic data capture), Medidata Classic Rave ® v2019.2.0<br>Nextera® Rapid Capture Enrichment (Illumina); HiSeq™ 2500, HiSeq™ v4, NovaSeq™, HiSeq™ X, or HiSeq™ 4000 (Illumina)<br>Navios Cytometer (Beckman Coulter Life Sciences)
Data analysis	SAS® Proprietary Software v9.4, SAS Institute Inc., 2002-2012<br>Seurat R v4.0.2 and v4.1.0, ggplot2_3.3.5, and ggpubr_0.4.0<br>GSNAP v2013-10-10, R v4.0.5 (2021-03-31), xCell (v1.3), Strelka (v1.0.14), and Lofreq (v2.1.2), Ensembl Variant Effect Predictor (v77)<br>Navios Analysis Software version 2.1 (Beckman Coulter Life Sciences)<br><br>The custom code used to generate the IMMUNOME results reported in this paper can be accessed at https://doi.org/10.5281/zenodo.6811671.

For manuscripts utilizing custom algorithms or software that are central to the research but not yet described in published literature, software must be made available to editors and reviewers. We strongly encourage code deposition in a community repository (e.g. GitHub). See the Nature Research guidelines for submitting code & software for further information.

Data

Policy information about availability of data

All manuscripts must include a data availability statement. This statement should provide the following information, where applicable:

- Accession codes, unique identifiers, or web links for publicly available datasets
- A list of figures that have associated raw data
- A description of any restrictions on data availability

Complete de-identified patient data will be available indefinitely within 2 years after the last patient's last Survival Follow-Up visit. Qualified researchers may request access to individual patient-level clinical data through Vivli (data request platform used at the time of this writing): https://vivli.org/ourmember/roche/.
For up-to-date details on Roche's Global Policy on the Sharing of Clinical Information and how to request access to related clinical study documents, see https://go.roche.com/data_sharing.
Anonymized records for individual patients across more than one data source external to Roche cannot, and should not, be linked due to a potential increase in the risk of patient re-identification.
Requests for the exploratory biomarker data underlying this publication should be directed to LCMC3_Core_Study_Team@gene.com for consideration.

Data from Genome Reference Consortium Human Build 38 can be accessed at www.ncbi.nlm.nih.gov/assembly/GCF_000001405.26/.

Field-specific reporting

Please select the one below that is the best fit for your research. If you are not sure, read the appropriate sections before making your selection.

☒ Life sciences ☐ Behavioural & social sciences ☐ Ecological, evolutionary & environmental sciences

For a reference copy of the document with all sections, see nature.com/documents/nr-reporting-summary-flat.pdf

Life sciences study design

All studies must disclose on these points even when the disclosure is negative.

Sample size

An MPR rate of ≥15% was selected as evidence of clinical efficacy based on a prior study (Chaft, JE, et al. J Thorac Oncol. 11, 537-544 [2016]). To provide 95% power to detect a 10% difference (null hypothesis 5%) at a one-sided significance level of 0.05, we targeted 180 patients for enrollment.

Data exclusions

For the clinical data analyses, patients whose tumors had EGFR or ALK alterations were excluded per protocol. At the time that the protocol for the LCMC3 study was being drafted, data on the effect of EGFR mutations/ALK alterations on immune checkpoint inhibitors were only beginning to emerge. In addition, all of those data derived from the metastatic setting. The effect of PD-(L)1 therapy on early-stage disease in patients with EGFR mutations had not yet been studied.

For the IMMUNOME analyses, marker combinations detected in <50% of patients were excluded for two reasons. First, the difference in I-index that was used for feature pre-selection may have been difficult to approximate in cases where we had two orthogonal immunomes. In such cases, the index would be close to 1, and as we measured changes in the index with a single feature removed, numerical issues would have possibly been introduced. Second, in the model selection procedure, we only used presence/absence for features with low prevalence. Inclusion of features present in only a small fraction of individuals would have allowed for fitting pathological models in which a feature would be an indicator in only a few samples. By enforcing the median as non-zero, a given a feature is expected to be an indicator in at least half of the samples.

Cells with impossible cell surface marker combinations (e.g., both CD3+ and CD19+) were also excluded.

Replication

IMMUNOME analyses were performed independently in a CLIA-certified clinical laboratory environment. The high-quality laboratory standards included daily flow cytometer calibrations and weekly calibrations across the 8 separate flow cytometers. Technical replicates were therefore not needed. A validated dry down cocktail tube (DURA Innovations) and automated pipetting were used. Every 6 months, a bias evaluation is performed on the same cells on all instruments to confirm instrument-to-instrument reproducibility and bias. We also employed a 14-tube panel with repetitions of markers in several tubes to further ensure reproducibility.

Randomization

None (single-arm study).

Blinding

None (open-label study).

Reporting for specific materials, systems and methods

We require information from authors about some types of materials, experimental systems and methods used in many studies. Here, indicate whether each material, system or method listed is relevant to your study. If you are not sure if a list item applies to your research, read the appropriate section before selecting a response.

Materials & experimental systems

n/a	Involved in the study
☒	☐ Antibodies
☒	☐ Eukaryotic cell lines
☒	☐ Palaeontology and archaeology
☒	☐ Animals and other organisms
☐	☒ Human research participants
☐	☒ Clinical data
☒	☐ Dual use research of concern

Methods

n/a	Involved in the study
☒	☐ ChIP-seq
☐	☒ Flow cytometry
☒	☐ MRI-based neuroimaging

Human research participants

Policy information about studies involving human research participants

Population characteristics

Participants were ≥18 years old, had pathologically documented stage IB–IIIB NSCLC per the American Joint Committee on Cancer Staging System (8th edition), and were deemed surgically resectable and functionally operable by the treating physicians. Patients had disease that was measurable per Response Evaluation Criteria in Solid Tumors (RECIST; version 1.1) and an Eastern Cooperative Oncology Group performance status score 0–1.

Demographic characteristics are presented in Table 1. During the building of the IMMUNOME-based predictive model for MPR, gender, smoking status/history, tumor histology, nodal status, and PD-L1 expression were not identified as covariates. The AUC values for models based on IMMUNOME data only or IMMUNONE data plus one clinical variable are presented in Table S6.

Recruitment

The LCMC3 study was conducted at academic institutions throughout the US. Each site implemented screening protocols to identify and offer enrollment to patients with medically operable and technically resectable NSCLC in thoracic surgery and medical oncology clinics. Given the neoadjuvant nature of the study, most patients were enrolled prior to knowledge of predictive biomarkers utilized in more advanced disease stages, making any enrollment selection bias unlikely. As shown in Table 1, the smoking status, histology, and PD-L1 expression profile of this study population parallel that of a typical population of patients with NSCLC. In review of the data, we did not appreciate any enrollment biases or imbalances that would have impacted the study results.

Ethics oversight

The study was conducted in compliance with the Declaration of Helsinki and International Conference on Harmonization Guidelines for Good Clinical Practice and was approved by the institutional review board at each participating site (Washington University School of Medicine, St. Louis, MO; New York University, New York, NY; The Ohio State University, Columbus, OH; Karmanos Cancer Institute, Detroit, MI; Brigham and Women's Hospital and Dana-Farber Cancer Institute, Boston, MA; City of Hope Comprehensive Cancer Center, Duarte, CA; Moffitt Cancer Center, Tampa, FL; UCLA Community Oncology Practice, Los Angeles, CA; Dartmouth-Hitchcock Medical Center, Lebanon, NH; University of Colorado Cancer Center, Denver, CO; Memorial Sloan-Kettering Cancer Center, New York, NY; Winship Cancer Institute, Emory University School of Medicine, Atlanta, GA; and Yale Cancer Center, New Haven, CT). All patients provided written informed consent.

Note that full information on the approval of the study protocol must also be provided in the manuscript.

Clinical data

Policy information about clinical studies

All manuscripts should comply with the ICMJE guidelines for publication of clinical research and a completed CONSORT checklist must be included with all submissions.

Clinical trial registration

NCT02927301

Study protocol

The study protocol is available at: https://www.clinicaltrials.gov/ct2/show/study/NCT02927301.

Data collection

The study was performed at 13 sites in the United States, specifically Washington University School of Medicine, St. Louis, MO; New York University, New York, NY; The Ohio State University, Columbus, OH; Karmanos Cancer Institute, Detroit, MI; Brigham and Women's Hospital and Dana-Farber Cancer Institute, Boston, MA; City of Hope Comprehensive Cancer Center, Duarte, CA; Moffitt Cancer Center, Tampa, FL; UCLA Community Oncology Practice, Los Angeles, CA; Dartmouth-Hitchcock Medical Center, Lebanon, NH; University of Colorado Cancer Center, Denver, CO; Memorial Sloan-Kettering Cancer Center, New York, NY; Winship Cancer Institute, Emory University School of Medicine, Atlanta, GA; and Yale Cancer Center, New Haven, CT. Patients were enrolled between April 20, 2017, and February 3, 2020.

Outcomes

The primary endpoint was major pathologic response (MPR; ≤10% viable malignant cells per local pathology assessment) in the primary tumor at resection. MPR was assessed locally per study-specific pathology training and standard operating procedures and subsequently reviewed by a central pathology committee.

Secondary endpoints included investigator-assessed objective response rate by RECIST, pathologic complete response, pathologic response by PD-L1 expression and tumor mutational burden, and safety (per Common Terminology Criteria for Adverse Events, v4.0). PD-L1 status was centrally determined by immunohistochemistry using the DAKO PD-L1 (22C3) assay.

Exploratory endpoints for patients with an MPR assessment included disease-free survival (time from complete resection to disease

recurrence or death from any cause) and overall survival (time from first atezolizumab dose to death from any cause). Correlative analyses included paired exome sequencing of tumor and blood DNA, peripheral blood immunophenotyping (IMMUNOME), and RNA sequencing (RNAseq) and single-cell RNAseq of tumor samples.

Flow Cytometry

Plots

Confirm that:

☒ The axis labels state the marker and fluorochrome used (e.g. CD4-FITC).

☒ The axis scales are clearly visible. Include numbers along axes only for bottom left plot of group (a 'group' is an analysis of identical markers).

☒ All plots are contour plots with outliers or pseudocolor plots.

☒ A numerical value for number of cells or percentage (with statistics) is provided.

Methodology

Sample preparation	A total of 6 mL of peripheral whole blood was collected in K3 EDTA tubes, which were inverted 8–10 times and shipped at ambient temperature (15–25°C).
Instrument	Navios Cytometer (Beckman Coulter Life Sciences)
Software	Navios Analysis Software version 2.1 (Beckman Coulter Life Sciences)
Cell population abundance	Sequential gating was utilized to ensure that the correct population of cells was analyzed. Viable cells (debris gate), side scatter vs. forward scatter, and CD45 vs. side scatter were used to identify lymphocytes. The first tube of the panel (Lymphosum) was used to enumerate T cell, B cell and NK cell fractions within the lymphocyte gate, totaling 95-100% with less than 5% myeloid contamination (CD13/CD14) to verify that the markers were used on the correct cell populations. Post-sort fractions were not analyzed.
Gating strategy	Plots were gated using three different strategies: LIN– total, LIN– CD11b+, and LIN– CD33+. Tube 13 (senescent cells) used both lymphocyte and sequential gating to find the immune cell population of interest. Lymphocyte gating isolated CD28– CD16– CD56– CD3+ cells, which were sequentially gated on CD57. Events falling with the CD57+ region were considered senescent cells. Senescent cells were further classified into subsets defined by positivity and negativity for CD4, CD8, KLRG1, and CD127. Tube 14 (dendritic cells) used LIN as an exclusion gate. Plots were gated using three different strategies: LIN– total, LIN– CD1c+, and LIN– CD141+.

☒ Tick this box to confirm that a figure exemplifying the gating strategy is provided in the Supplementary Information.
